# Expression dynamics of repetitive DNA in early human embryonic development

**DOI:** 10.1186/s12864-019-5803-1

**Published:** 2019-05-31

**Authors:** Cihangir Yandım, Gökhan Karakülah

**Affiliations:** 1 İzmir Biomedicine and Genome Center (IBG), 35340, İnciraltı, İzmir, Turkey; 2 Department of Genetics and Bioengineering, İzmir University of Economics, Faculty of Engineering, 35330, Balçova, İzmir, Turkey; 3 Department of Medicine, Division of Brain Sciences, Hammersmith Hospital, Imperial College London, Faculty of Medicine, W12 0NN, London, UK; 4 İzmir International Biomedicine and Genome Institute (iBG-İzmir), Dokuz Eylül University, 35340, İnciraltı, İzmir, Turkey

**Keywords:** Repetitive DNA, Human development, Pre-implantation, Single cell RNA sequencing, Repeatome, Bioinformatics, WGCNA, Satellite repeats, DNA methylation, Repeat enrichment

## Abstract

**Background:**

The last decade witnessed a number of genome-wide studies on human pre-implantation, which mostly focused on genes and provided only limited information on repeats, excluding the satellites. Considering the fact that repeats constitute a large portion of our genome with reported links to human physiology and disease, a thorough understanding of their spatiotemporal regulation during human embryogenesis will give invaluable clues on chromatin dynamics across time and space. Therefore, we performed a detailed expression analysis of all repetitive DNA elements including the satellites across stages of human pre-implantation and embryonic stem cells.

**Results:**

We uncovered stage-specific expressions of more than a thousand repeat elements whose expressions fluctuated with a mild global decrease at the blastocyst stage. Most satellites were highly expressed at the 4-cell level and expressions of ACRO1 and D20S16 specifically peaked at this point. Whereas all members of the SVA elements were highly upregulated at 8-cell and morula stages, other transposons and small RNA repeats exhibited a high level of variation among their specific subtypes. Our repeat enrichment analysis in gene promoters coupled with expression correlations highlighted potential links between repeat expressions and nearby genes, emphasising mostly 8-cell and morula specific genes together with SVA_D, LTR5_Hs and LTR70 transposons. The DNA methylation analysis further complemented the understanding on the mechanistic aspects of the repeatome’s regulation per se and revealed critical stages where DNA methylation levels are negatively correlating with repeat expression.

**Conclusions:**

Taken together, our study shows that specific expression patterns are not exclusive to genes and long non-coding RNAs but the repeatome also exhibits an intriguingly dynamic pattern at the global scale. Repeats identified in this study; particularly satellites, which were historically associated with heterochromatin, and those with potential links to nearby gene expression provide valuable insights into the understanding of key events in genomic regulation and warrant further research in epigenetics, genomics and developmental biology.

**Electronic supplementary material:**

The online version of this article (10.1186/s12864-019-5803-1) contains supplementary material, which is available to authorized users.

## Background

A fine-tuned orchestration in genome regulation drives the transition between totipotency and pluripotency within the first few rounds of cell divisions following fertilisation in mammals. This process starts with the erasure of DNA methylation as well as most chromatin marks and re-establishing them gradually; a phenomenon known as “epigenetic reprogramming” [1]. During reprogramming, the epigenetic asymmetry between maternal and paternal genomes is equalised and epigenetic organisation takes place to give rise to lineage specific gene expression [1–3]. Importantly, the cascades of nuclear events during this period are also imperative in terms of building a de novo chromatin architecture, which is essential for the development of a healthy embryo [4].

A key hub for chromatin architecture lies within centromeres and pericentromeres, where densely packed nucleosomes contribute to constitutive heterochromatin. Decorated with a multitude of epigenetic marks; constitutive heterochromatin at pericentromeres provides a solid basis for the formation of functional centromeres and kinetochores [5]. Interestingly, two crucial heterochromatin marks; H4K20me3 and H3K64me3 are erased from the maternal genome shortly after the first cell division [6, 7] and the substantial heterochromatin mark; H3K9me3 gets passively diluted until the fourth division takes place [8, 9]. This causes a rather relaxed heterochromatin configuration especially at pericentromeres [10]. This atypical heterochromatin conformation conceivably accommodates the necessary platform for successful reprogramming and has been associated with nucleolar-like bodies (NLBs) that resemble rings, which are visible until the third division [11–14]. At 4-cell stage, during which H3K9me3 levels are significantly low [9, 15], NLBs start to be replaced with chromocentres; precursors of typically dense somatic heterochromatin [9, 13–16]. Even though most NLB to chromocentre transitions were reported in mouse development, similar features were also observed for human embryos [17].

Heterochromatin formation is an indispensible step in building a brand new chromatin [18, 19] and it is known to be triggered by the expression of pericentromeric satellite repeats in mouse [19–22]. Local RNAs formed due to the expression of these tandem repeats were shown to recruit the heterochromatin protein HP1α; resulting in de novo heterochromatin formation [23, 24]. This finding not only provides an insight into the mechanism of chromatin organisation during mammalian pre-implantation development but also highlights the significance of repetitive DNA, which is often overlooked. Indeed, more than half of the human genome consists of various repetitive DNA sequences [25, 26] but there has been a paucity of information on the expression profiles of repeat elements despite the overwhelming number of RNA sequencing (RNA-seq) studies reported on various biological contexts. This could be attributed to the alignment problems of repetitive DNA using classical gene expression pipelines. Hence, the contribution of repetitive DNA expression to genomic regulation and architecture has not been revealed sufficiently. On the other hand, reported links between aberrant repeat transcription and cancer [27–30] as well as other diseases including autoimmune disorders [31, 32] brought out the importance of this actuality to genomic control and human physiology; with the caveat that the mechanisms rendering repeat expression influential in these contexts are poorly understood.

Several studies reported expression differences across human pre-implantation stages for a limited number of repeats. HERV-K (flanking with LTR5_Hs) elements were shown to be expressed at their highest level at the morula stage, whereas HERV-H elements reached their peak level in embryonic stem cells [33, 34]. On the other hand, SINE-VNTR-Alu (SVA) repeats were shown to be expressed at high levels at 8-cell and morula stages [33]. A more recent study provided a cross-species comparison and confirmed these results [35]. However, none of these aforementioned studies yet reported a systematic analysis on all subtypes of all repeat families. Importantly, there was still no information on the embryonic regulation of satellite repeats; which were shown to be essential for establishing de novo chromatin architecture in early mouse embryos [19–22]. Likewise, regulatory effects of all repeats on the expression profiles of development specific genes, which are located nearby repeats, have also not been elucidated. Finally, the contribution of repeats to the lineage specification of the genome during the early stages of cell fate allocation is unknown. Understanding the levels of cell-to-cell variation in the expressions of repeat elements across single cells would help to characterise the dynamic genomic behaviour as cellular differentiation takes place.

To address these issues and examine the human pre-implantation development from a repeats perspective at the global scale, we aimed to uncover the expression dynamics of the whole repeatome in this early phase of human embryos. To realise this aim, we analysed a previously published and publicly available dataset, where total RNA from 124 single cells from different stages of early human development were sequenced to uncover non-coding transcripts [36]. This RNA-seq data was obtained from all pre-implantation stages from oocyte to late-blastocyst level as well as passage-0 (P0) and passage-10 (P10) embryonic stem cells (ESCs) derived from the inner cell mass (ICM) of blastocysts. Importantly, single cells used to generate this dataset were originally obtained from at least two different embryos for each pre-implantation stage. It is also worth mentioning that the dataset used in this study is -to our knowledge- the only published dataset that allows one to study total non-coding satellite transcripts in human pre-implantation single cells. Other published studies either make use of this same dataset or use poly (A) pre-selection in their laboratory procedures to focus on gene-arisen mRNAs or a limited number of transposable elements [33, 37–40].

## Results

### The repeatome exhibits distinct expression patterns across stages of human pre-implantation

**Fig. 1:**
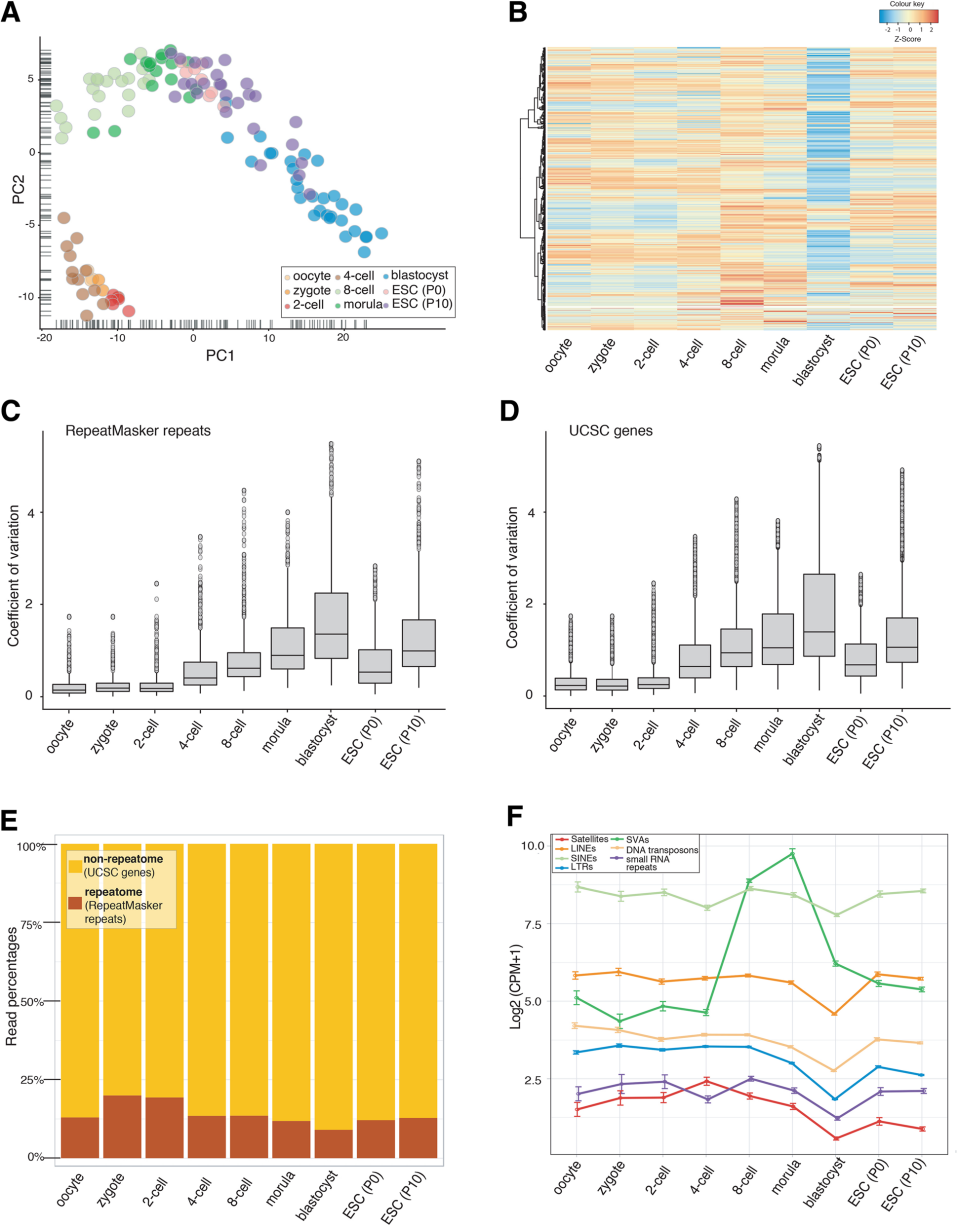
Distinct expression patterns of repeatome across the stages of human pre-implantation and in ICM-derived embryonic stem cells (ESCs). **a** Principal component analysis (PCA) of repeatome’s expression levels. Each circle represents a single cell in the corresponding developmental stage. **b** Heat-map representation of the expression levels of all 1116 repeat elements. Each row represents a single repeat and columns represent developmental stages. **c** Box-plot representation of the coefficients of variation (CV) in repeat expression. **d** Box-plot representation of the CVs in gene expression. **c-d** CVs across all single cells in a given developmental stage were calculated for each individual repeat element or gene separately and they were plotted as boxes and whiskers. **e** Read percentages of repeat-arisen transcripts against all gene-arisen transcripts. Read percentages were calculated from the ratio of the number of all RepeatMasker annotated repeat transcripts over the total transcript number, which is the sum of all repeat-arisen transcripts and the transcripts originated from UCSC annotated genes. **f** Average expression levels of different repeat classes given as log2[Counts Per Million (CPM + 1)]. Error bars represent the standard error of the mean. [For ESCs; P0:passage 0 and P10:passage10]

There are more than a thousand types of repeat elements identified within the human genome [26, 41]. We performed a holistic analysis for the expression of these elements by applying the Repenrich2 pipeline [27] and analysed the expressions of 1116 repeats. Remarkably, the principal component analysis (PCA) showed that the expression levels of repeats were clustered in a distinguishable manner across different stages of pre-implantation; in line with the PCA analysis of the gene expression apart from the fact that blastocysts and ESCs were clustered separately in the latter one (Fig. 1a and b, Additional file 1: Figure S1). Additional file 2: Table S1 summarises the number of data points for each group. The PCA analysis highlights the fact that distinct expression patterns throughout human pre-implantation stages are not only exclusive to genes and long non-coding RNAs, but repetitive DNA is also expressed in a coordinated manner in this biological context [36, 42]. PCA analysis for repeats revealed two major clusters. Oocytes, zygotes, 2- and 4- cell embryos clustered at the bottom side, whereas 8-cell embryo, morula and blastocysts as well as ESCs clustered at the upper side of the plot. The most dramatic difference in the expression profiles was between 4- and 8-cell stages; agreeing well with the gene expression analysis reported before with the same plot [36]. It is intriguing to note that this time-frame also coincides with the major embryonic genome activation [40, 43–45]. Other interesting points were that ESCs clustered more closely with morula cells rather than blastocyst cells **(**Fig. 1a**)** and that blastocyst cells exhibited a decrease in the z-scores for the transcription of repeats **(**Fig. 1b**)**. This could be attributed to a mild decrease in the expression of repeats at the blastocyst stage as shown in Additional file 1: Figure S2. To our knowledge, this is the first time where the expression of the whole repeatome was shown to display a coordinated trend during early human development.

One of the important research questions in pre-implantation development is the timing and mechanism of lineage specification. Conventionally, it is accepted that lineage specific gene expression starts at the 8-cell stage in mouse [13, 46–49] but some reports suggest that molecular events needed for lineage commitment start during 4-cell stage [47, 48, 50, 51]. To see the timing of diversification in repeat element expression across single cells, we plotted coefficients of variation (CV), as described before [52]. Interestingly, there was a notable increase in the variation of repeat expression levels at 4-cell stage compared to 2-cell stage and this variation was elevated gradually as the cell divisions continued **(**Fig. 1c**).** The variation in gene expression levels also showed the same trend with the marked difference between 2- and 4-cell stages (Fig. 1d). One-way ANOVA followed by Tukey’s multiple comparison tests was conducted to determine the statistical significances of the differences observed for each particular developmental stage with any other stage. According to this, there was a statistically significant (*p*-adj < 0.001) difference in the CVs for repeat elements between all stages except the fact that there was no difference between oocyte, zygote and two cell stages as well as between eight cell and ESC-P0. A similar result on the statistical significance was obtained for the CVs of genes; however, this time there was no difference between 4-cell stage and ESC-P0.

In addition to this, relative percentages of repeat-arisen transcripts with respect to gene-arisen transcripts were higher at zygote and 2-cell stages (Fig. 1e). When we looked at the expression levels of different repeat families, we realised that satellite expression was emphasised at the 4-cell level (Fig. 1f, Additional file 1: Figure S3). However, not all satellites behaved the same way (Additional file 1: Figure S4). On the other hand, SINEs (Short Interspersed Nuclear Element) and small RNA repeats exhibited a decrease in their expressions at the 4-cell stage. In addition to this, all members of repeat families except the SINE-VNTR-Alu (SVA) elements exhibited a decrease in expression at the blastocyst stage (Fig. 1f, Additional file 1: Figure S3). As reported before, expressions of SVA elements were clearly increased at 8-cell and morula stages [33, 38]. LTR (Long Terminal Repeat) expression was slightly increased at 8-cell and morula, as was the expression of LINE (Long Interspersed Nuclear Element) elements even though the latter was less pronounced. The count numbers, fold changes and FDR (False discovery rate) analyses for repeats and genes could be found in the Additional file 3: Table S2, Additional file 4: Table S3 Additional file 5: Table S4, Additional file 6: Table S5, Additional file 7: Table S6.

### Expression dynamics of satellites

**Fig. 2:**
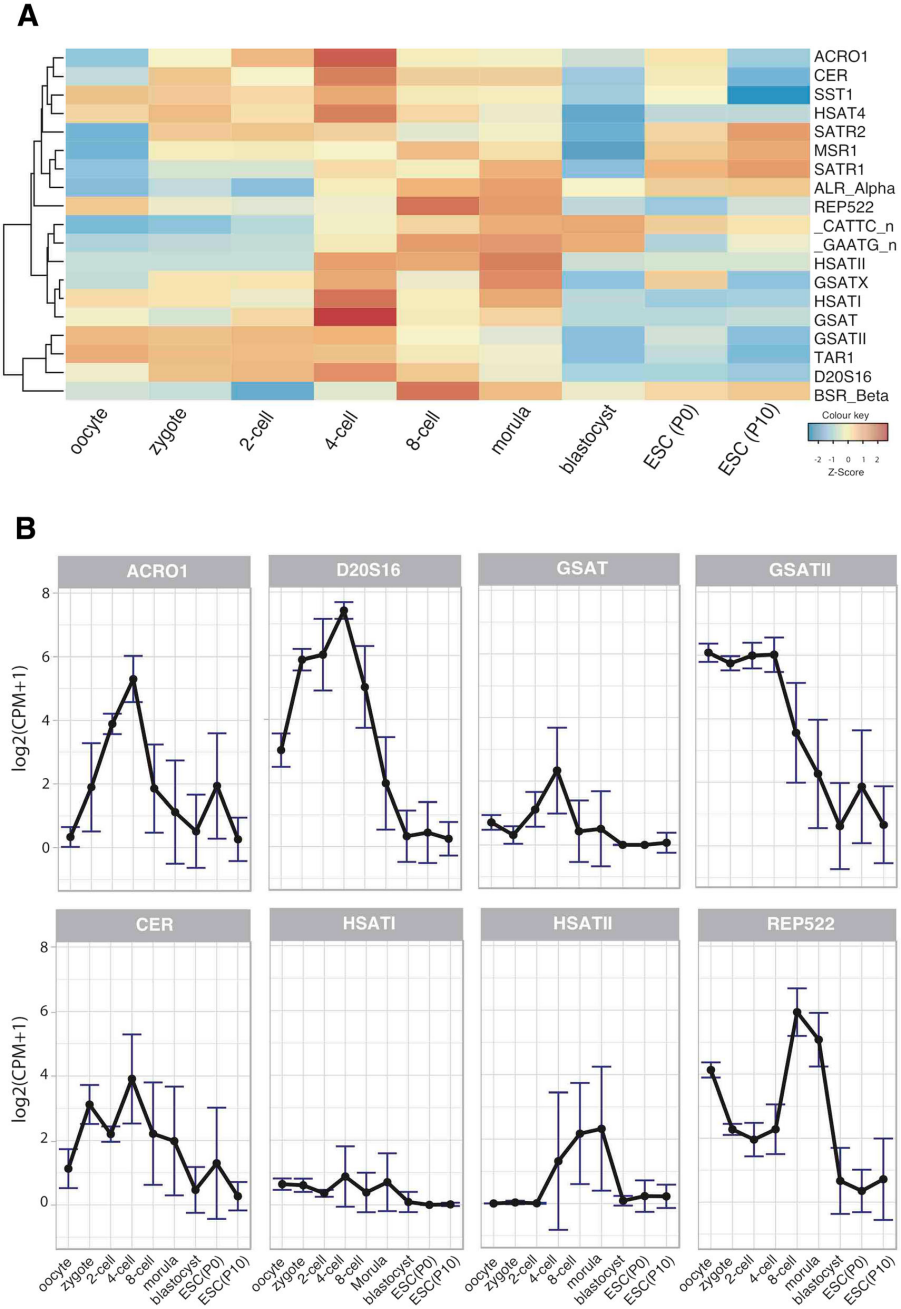
Expression dynamics of satellite repeats. **a** Heat-map representation of satellite expressions. Each row represents a single repeat and columns represent developmental stages. **b** Line-plots of individual satellites, which exhibit high expression levels at 4-cell (ACRO1, D20S16, GSAT, GSATII and CER) and 8-cell (REP522) stages. HSATI and HSATII expressions are also shown. Each data point represents the average expression level [log2(CPM + 1)] of the particular repeat in single cells. Error bars represent the standard error of the mean. See Additional file 1: Figure S4 for line plots of all members of the satellite family

Satellites are repeats that are predominantly found in tandem arrays, whose repeating unit could range from less than a 100 bp to approximately 1500 bp. Even though they are mostly found at centromeres and pericentromeres, some of them are located at various chromosomal regions including intergenic islands as well as telomeres [53]. Our expression analysis using the RepEnrich2 pipeline revealed that most of the satellites were expressed during human pre-implantation with stage specific patterns [27]. A number of satellite elements were highly expressed during 4-cell stage (Fig. 2a). Yet some other elements were not expressed (i.e. HSAT6 and SAR) or hardly expressed (i.e. Subtel_Sa) during pre-implantation stages or in ESCs (Additional file 1: Figure S4).

Among those that are expressed, the expression levels of ACRO1 and D20S16 peaked at 4-cell stage (Fig. 2a and b). ACRO1 is a 147 bp satellite repeat found in short arms of acrocentric chromosomes nearby nucleolus organiser regions [54], which contain ribosomal RNA genes and are essential components of chromatin architecture [55]. D20S16 satellite repeats, which are formed by 98 bp dimers [56], also showed a similar expression trend as ACRO1. It is as well noticeable that the expression of predominantly centromeric CER and mostly pericentromeric GSAT satellite repeats peaked at 4-cell level even though this is less pronounced compared to the trend seen for ACRO1 and D20S16 repeats. GSATII was expressed at high levels until the third cell division (end of 4-cell stage) and showed a steep decrease in the following divisions. Moreover; HSATI and HSATII, which were shown to be involved in human cancers [28, 29], were variably expressed within the window that covers 4-cell, 8-cell and morula stages and were hardly expressed in other time-frames (Fig. 2a and b). On the other hand, REP522, which is found mostly in telomeric regions, was upregulated at 8-cell level. Expression line graphs for all other satellite repeats could be seen in the Additional file 1: Figure S4. These results for the first time uncover the satellite expression dynamics across human pre-implantation development stages. Expressions of ACRO1 and D20S16 seemed to clearly peak at the 4-cell stage. This time-frame notably coincides with the emergence of de novo heterochromatin in mouse [13, 17, 19, 21].

### Expression dynamics of transposons and small RNA repeats

**Fig. 3:**
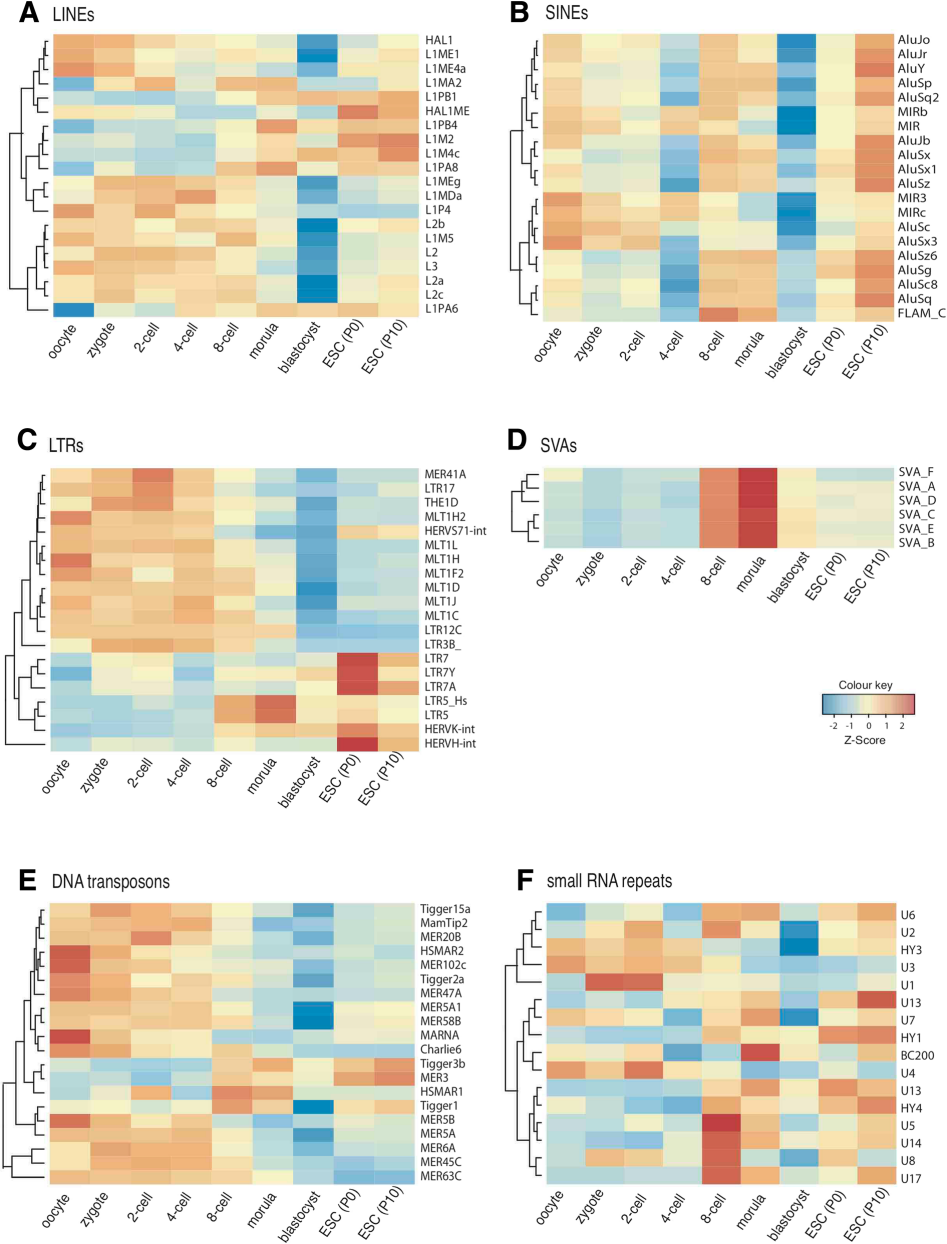
Expression dynamics of transposons and small RNA repeats. Heat-maps represent expression levels of top 20 most-variable repeats for (**a**) LINEs, (**b**) SINEs, (**c**) LTRs, (**e**) DNA transposons, (**f**) small RNA repeats; and all members of (**d**) SVA elements. Each row represents a single repeat and columns represent developmental stages

In order to identify crucial transposons and small RNA repeats in pre-implantation cells and ESCs, we drew heat-maps for top-variable 20 repeat elements for each of these repeat families. Our results are in line with previous studies, which reported LINE and SINE expression in human pre-implantation [37, 38]. Some LINE elements were expressed more in earlier stages of pre-implantation whereas others showed elevated expression z-scores at later stages (Fig. 3a). These include different members of L1, L2 and L3 families. In addition, top variable SINE elements showed a decrease in their z-scores at 4-cell and blastocyst levels globally (Fig. 3b). They seem to reach higher values in oocytes and ESCs though. A member of SINE family; FLAM_C, showed an increase at the 8-cell level.

Recently, LTR transposons have gained attention in pre-implantation development and numerous reports showed their expression in this context [33, 34, 37, 38, 40, 49, 57–60]. Transcription of HERVK-int and HERVH-int as well as LTR5_Hs was reported to be of particular importance [34, 35, 37, 40]. Our LTR expression analysis was in agreement with these previously published studies; demonstrating an increased z-score in the expression for HERVK-int, HERVH-int and LTR5_Hs elements at later stages of pre-implantation (Fig. 3c). Together with LTR5_Hs, LTR5 also showed a stage-specific increase in 8-cell and morula cells. HERVK-int and HERVH-int showed their highest z-scores in ESCs passage 0; accompanied by a marked increase in LTR7, LTR7Y and LTR7A. On the other hand, numerous other elements including members of the MLT1 family had higher expression z-scores in the initial stages of pre-implantation.

SVA repeats, which are non-LTR retrotransposons, exhibited an increase at 8-cell and morula stages (Fig. 3d), again in concordance with previously published studies [37, 38, 40]. Members of DNA transposon family, on the other hand, were expressed mostly in oocytes and early stages of human pre-implantation with some exceptions (e.g. MER3, HSMAR1), which showed higher expression z-scores in later stages (Fig. 3e). Our analysis also pointed out some small RNA repeats with increased expression levels in a stage specific manner (Fig. 3f). A higher level of U1 spliceosomal RNA expression, which was associated with mouse development before [61–63], was linked to zygote and 2-cell stages in our analysis. Yet, expression z-score of 5S RNA was also increased at these stages. Spliceosomal U5 RNA and a few other small RNAs, U8, U14 and U17, whose functions were associated with rRNA formation [64, 65], had higher expression z-scores in the 8-cell embryo. Moreover, the expression of BC200 small RNA, which was shown to play important roles in brain development [66], was highest at the morula stage.

### Repeat elements and the expression of nearby genes

**Fig. 4:**
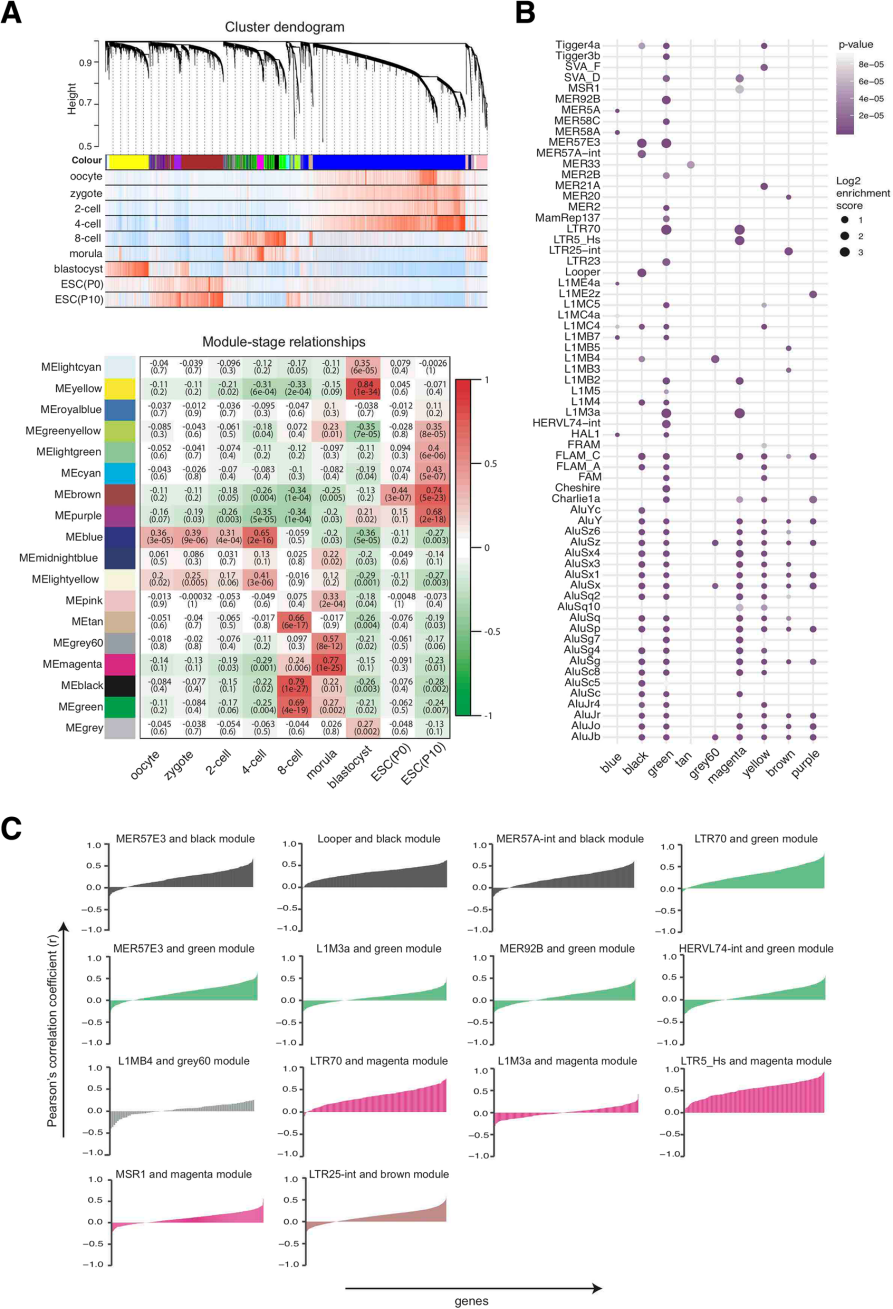
Enrichments of repeat elements in the 2-kb upstream promoter regions of stage-specific genes and expression correlations of enriched repeats with these genes. **a** Weighted Gene Co-expression Network Analysis (WGCNA) of human pre-implantation development stages and ESCs. Upper panel shows the cluster dendrogram with hierarchical clustering of co-expressed gene clusters (modules), which correspond to long branches in the dendrogram and are represented as specific colours underneath each branch. Vertical lines below module specific colours represent the correlation statuses of genes with a particular developmental stage (red: positive correlation, blue: negative correlation). Lower panel shows module-stage relationships. Rows correspond to module eigengenes and columns to human pre-implantation stages and ESC passages. The Pearson’s r correlation coefficients and associated *p*-values are given in each cell. The colour code indicates the degree of correlation (red: high, green: low). See Additional file 1: Figure S5 for expression heat-maps of module-specific genes as well as Gene Ontology analysis results for these genes. **b** Dot-plot presenting the enrichment levels of repeats within the 2 kb-upstream promoter regions of genes listed in the corresponding modules. Stage-specific modules were given in columns and repeat elements that exhibit enrichment were given in rows. The radii of dots represent ES and the colour intensity indicates *p*-values obtained by Fisher’s exact test. **c** Expression correlations of module-enriched repeats with genes in the corresponding module. The Pearson’s correlation coefficients (r) were calculated using all expression data points from single cells from all developmental stages and ESCs for each enriched repeat (ES > 3.5) and all genes in given modules individually. R correlation coefficients obtained for a particular repeat element and each single gene in the relevant module were presented as stacked columns. Column colours indicate specific repeat-enriched modules (e.g. green colour indicates genes within the 8-cell specific green module). Additional file 1: Figure S8 shows additional interesting repeat-module correlations where ES is less than 3.5 threshold

To see if there is a correlation between expression levels of repeats and nearby genes, we looked at the occurrence rates of repeat sequences in gene-sets that exhibit stage specificity in expression during pre-implantation and in ESCs. Firstly, we identified stage-specific gene expression networks using Weighted Gene Co-expression Network Analysis (WGCNA) [67, 68]. Our analysis revealed 18 distinct modules represented with different colours (Fig. 4a, upper and lower panel). Stage-specific genes follow the trend of overexpression in a single developmental stage. We focused on 9 modules, which in terms of gene expression exhibit stage specificity (r > 0.50, *p*-value < 10^− 3^) at a single pre-implantation stage (Fig. 4a, lower panel). Our Gene Ontology analysis [69] revealed that the context of genes in these stage-specific modules were in agreement with the previously reported [36, 42] functional classification of differentially expressed genes in human pre-implantation (Additional file 1: Figure S5, Additional file 8: Table S7). Next, we investigated the enrichment levels of repeats within the promoter regions of all genes in the aforementioned 9 stage-specific network modules. This involved counting the occurrence rates of individual repeat sequences in the 2-kb upstream regions of genes and calculating an enrichment-score (ES) against their occurrence rates in the whole reference genome alongside a Fisher’s exact test. We showed the results of this analysis on a dot-plot (Fig. 4b). Most of the enriched repeats fell into LINE (e.g. L1 family) and SINE (e.g. Alu elements) category, but the highest enrichment scores were obtained for LTR elements and DNA transposons. For repeats that showed a high level of enrichment (absolute enrichment score (ES) > 3.5, *p*-value < 10^− 3^) in a stage-specific module, we analysed r correlation coefficients between the expressions of the particular repeat element and all genes within the given module across all stages of pre-implantation and in ESCs. We plotted the r-coefficients for all genes in the specific module where a particular repeat is enriched (Fig. 4c).

Looper, a DNA transposon enriched in the 8-cell stage specific black module, had moderate levels of positive correlations with genes in this module whilst Looper itself being upregulated at the same particular pre-implantation stage (Additional file 1: Figure S6). Similarly, LTR70 expression was upregulated in the 8-cell and morula stages; its occurrence was enriched within the 2 kb upstream regions of genes in green (8-cell) and magenta (morula) modules (Fig. 4b); and its expression correlated positively up to moderate levels with genes listed in these modules (Fig. 4c). As an example, LTR70 expression correlated highly with the expression of a magenta module (morula) gene; *P32* (*C1QBP*), which was reported to be crucial in fetal development and mitochondrial translation (Additional file 1: Figure S7) [70]. LTR5_Hs, whose sequence occurrence was enriched in the magenta (morula) module, also showed meaningful levels of positive expression correlations with genes listed in this module; accompanied by an increase in the expression of LTR5_Hs per se in the morula stage (Additional file 1: Figure S6). Interestingly, LTR5_Hs expression correlated highly with the expression of the magenta (morula) module gene; *NANOGNB*, which has a highly regulatory function during pre-implantation (Additional file 1: Figure S7) [71]. In addition to these repeats, we realised that FLAM_C and SVA_D exhibited moderate to high levels of positive correlations with specific modules despite the enrichment levels of these two repeats were below the 3.5 ES threshold (Additional file 1: Figure S8). More specifically, the correlation of the expressions of FLAM_C and the magenta (morula) gene *CABP4* was noticeable (Additional file 1: Figure S7). CABP4 protein was shown to regulate eye development in mouse [72].

### DNA methylation dynamics of repeat elements

**Fig. 5:**
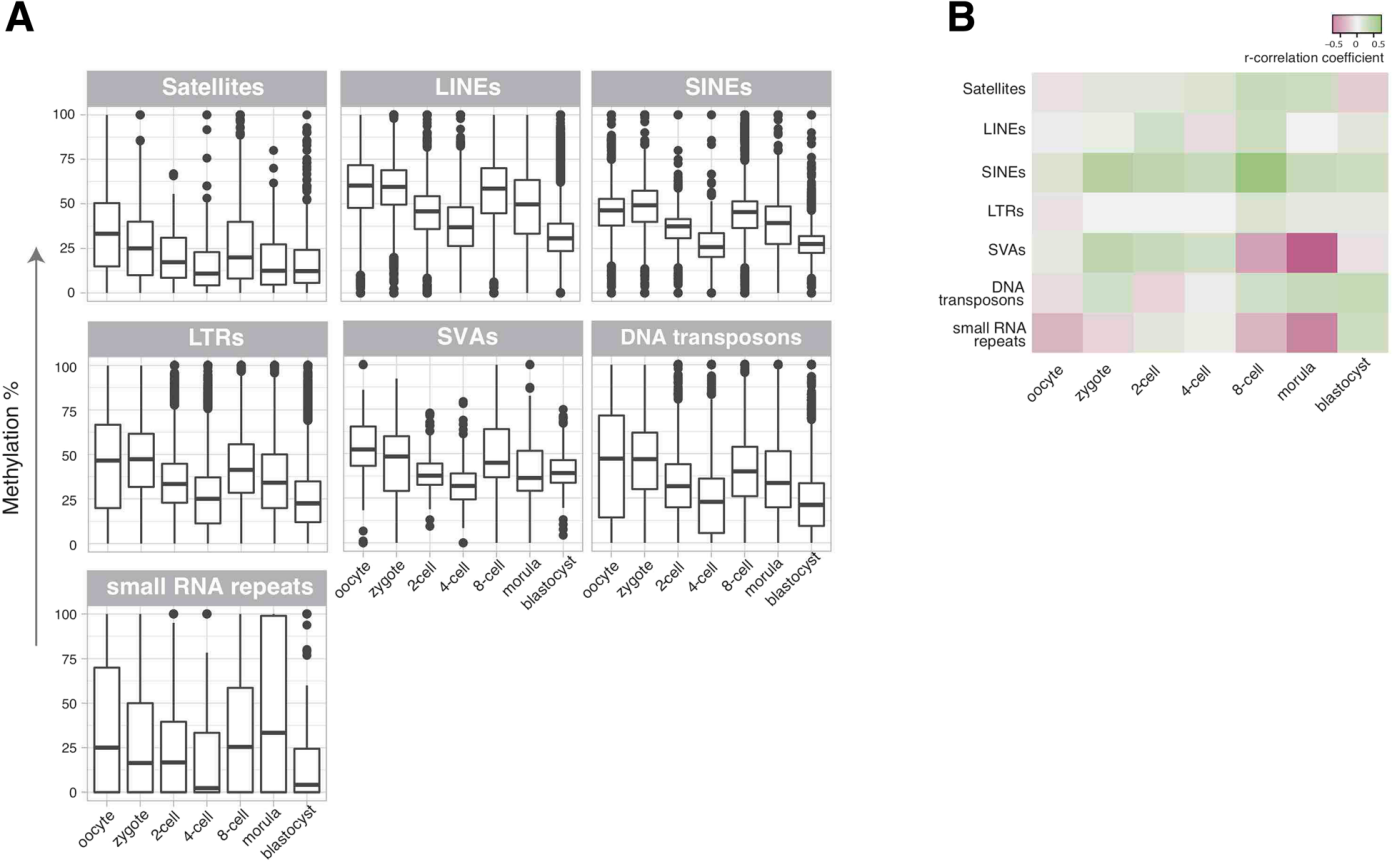
DNA methylation dynamics of major repeat classes during human pre-implantation. **a** Box-plot representations of DNA methylation levels from single cells across stages of pre-implantation categorised by repeat classes. **b** DNA-methylation and expression correlation heat-map. Each cell in the heat-map represents r-correlation coefficients between the average expression levels obtained from a single cell dataset [36] and average methylation levels for another single cell dataset [76] for all members of a given repeat class in the specified time-frame during pre-implantation

Even though the methylation of DNA and its effects on genomic regulation has been widely addressed in the context of early mouse and human development [13, 37, 59, 73–76], a complete picture that outlines how the repeatome gets affected by global methylation events during this period has still been missing. We analysed DNA-methylation levels of all repeats utilising another published dataset on single cells of human pre-implantation embryos [76]. The number of data obtained from single cells using multiple embryos is summarised in the Additional file 9: Table S8. The R code for this analysis was provided in the Additional file 10 and methylation percentages for all repeats were given in the Additional file 11: Table S9. Our analysis revealed a global dip in DNA methylation at the 4-cell level and a slight increase at the 8-cell level, in agreement with similar results reported before for global gene studies on pre-implantion development (Fig. 5a) [13, 37, 59, 76]. We revealed that satellites, SVAs, DNA transposons and small RNA repeats were also affected by the global dip in DNA methylation at 4-cell level but some outliers still exist. One-way ANOVA followed by Tukey’s multiple comparison tests was conducted to determine the statistical significances of the differences observed for each particular developmental stage with any other stage and this was given as a supplementary table (Additional file 12: Table S10).

To figure out the impact of DNA methylation on the expression levels of repeats in different developmental stages, we calculated Pearson’s r correlation coefficients for mean repeat expression and mean repeat methylation levels across developmental stages. Our result indicated moderate to high levels of negative correlation between DNA methylation and expression levels for SVA transposons and small RNA repeats at 8-cell and morula stages (Fig. 5b); possibly implying the importance of DNA methylation/demethylation mechanisms in the regulation of these particular repeats during these particular pre-implantation stages. However, we could not capture a high level of negative correlation between DNA methylation and repeat expression at the global level.

Though there was a trend that most satellites followed global DNA methylation patterns, fluctuations in their expressions could not easily be explained by their DNA methylation levels. For example, a dramatic increase was obvious for the expression of D20S16 satellites at the 4-cell level (Fig. 2b), but the decrease seen at the DNA methylation was not as robust (Additional file 1: Figure S9). Likewise, the sharp increase in the expression of REP522 satellites at 8-cell level (Fig. 2b) was not accompanied with DNA demethylation at this stage for this particular repeat. More examples such as GSAT could be counted among repeats whose expressions could not simply be explained by DNA demethylation.

## Discussion

Our analysis on single cell pre-implantation and embryonic stem cell RNA-seq data presents the information obtained from the whole repeatome in early human development. The PCA analysis (Fig. 1a) confirmed the stage-specific behaviour of repeatome during this developmental frame. Moreover, the coupled increase in the intercellular variation in the expressions of repeats as well as genes during the 2-cell to 4-cell transition (Fig. 1c and d) was interesting to note. Lineage specification is widely accepted to start after the 8-cell stage [13, 77] and some evidence was reported for cell-fate allocation for mouse pre-implantation at stages before the 8-cell level [78]. Based on our variation analyses on repeat and gene expressions, the contribution of repeatome to this phenomenon would be a valuable hypothesis to be tested with future experimental studies. Another interesting point was the relatively high number of repeat-arisen transcripts with respect to gene-arisen transcripts in zygote and 2-cell stages, where maternally inherited factors are still in charge [79]. On the other hand, global levels of repeat expressions were mildly decreased in the blastula stage whereas this trend was not marked at the genes level.

Satellites are mostly associated with heterochromatin [80, 81] and it is known that the heterochromatic histone mark H3K9 methylation gets passively diluted during the first two cell divisions after fertilisation as mentioned above [8, 13]. This could be one of the possible reasons behind the obvious global increase in satellite expression at the 4-cell stage. A specific increase in the expressions of major satellites was reported at the 2-cell stage in mouse [15, 19]. This was just before the chromocentre formation took place at the 4-cell stage, whereas our analysis with human embryos revealed that most of the increase in satellite expression is taking place at the 4-cell level. This could point out a possible timing difference between mouse and human embryos, as reported before in other key developmental concepts such as zygotic/embryonic genome activation [42, 45, 82]. It is not known which satellite transcripts participate to de novo heterochromatin formation mechanisms in human embryos but those that show significant upregulation at 2- and 4-cell levels (e.g. ACRO1, D20S16, GSATI, GSATII and CER) could be explored. Moreover, it is worth mentioning that our analyses on human pre-implantation cells did not pick up high levels of HSATI and HSATII transcription, which was previously linked to cancer [28, 29]. Furthermore, it would be of preference to cross-validate our findings on repeat expressions with other datasets and upcoming studies on the RNA-sequencing of human embryos without the poly(A) pre-selection would help regarding this matter as mRNA-biased protocols do not allow one to detect all types of repeats; particularly excluding the satellites [83].

Interspersed repeats such as LINEs, SINEs, LTRs, DNA transposons, and small RNA repeats exhibited diverse expression patterns among different members of the relevant group (Fig. 3). Some members of these groups were highly expressed in ESCs and were hardly expressed in pre-implantation stages. Moreover, all members of the SVA transposons were highly expressed in 8-cell and morula stages. The high number of spatiotemporal data points used in this study allowed us to examine the correlations between repeat expressions and the expression levels of nearby genes, helping us to further refine the links between proximal repeats and gene expression. Genes in the magenta (morula) module, which were associated with ribonucleoprotein complex formation, RNA processing as well as cellular metabolism in the morula stage (Additional file 1: Figure S5), appeared to correlate well with their promoter enriched repeats LTR70, LTR5_Hs, SVA_D and FLAM_C (Fig. 4c). Nevertheless, LTR70 and some other repeats were also associated with the green module in terms of expression correlations and enrichments. The potential of these repeats on influencing the expression of genes in the relevant modules could be tested with future experimental studies.

Our DNA methylation analysis showed that repeatome is affected by global methylation changes during pre-implantation and our results are complementing earlier studies [37, 76]. The increase in the average expressions of all satellites at the 4-cell level corresponds well with the reported global dip in DNA methylation at this stage. On the other hand, our analysis suggests that expression is probably influenced by further factors (e.g. histone modifications such as H3K9 methylation) for satellites, as small changes in their DNA methylation statuses could not simply explain drastic changes in their expressions. When other repeats (e.g. transposons) were considered, methylation levels only negatively correlated with expression in particular pre-implantation stages and only for particular repeats; again highlighting possible involvement of further factors such as histone modifications, which were reported to be essential for the silencing of many repeats [80, 84]. Still, a combinatorial code of different types of DNA methylation (i.e. 5mC:5-methylcytosine, 5hmC:5-hydroxymethylcytosine, 5fC:5-formylcytosine and 5caC:5-carboxylcytosine) on repeats was also reported [85]. It should be noted that our analysis utilised a dataset that did not distinguish different types of DNA methylation. Our RNA expression and DNA methylation correlation was performed using the mean values for each data point because the two datasets were not paired. Future studies could make use of newer techniques, which enable collecting both RNA sequencing and DNA methylation data from a single cell [86].

## Conclusions

Taken together, our results show that repetitive elements undergo coordinated developmental changes in their expression patterns. This may underlie a possible regulatory role for the repeatome in human pre-implantation development. The marked increase in the expressions of satellite repeats could be investigated with further experimental studies in order to comprehensively understand the organisation of heterochromatin in the human embryo. Additionally, the factors involved in the DNA methylation / de-methylation of particular repeats such as SVA repeats and small RNAs should be identified as these elements showed highest negative correlation between expression and DNA methylation. Other key elements identified in this study could also serve as a beneficial resource for future studies in the field of genome research, epigenetics and developmental biology. The research pipeline followed in this study could provide an example where repetitive DNA expression and its links to gene regulation are studied.

## Methods

### Data collection

A total of 124 raw single cell RNA-sequencing (RNA-seq) data of developing human embryos presented in a previous study [36] (GEO Accession: GSE36552) were downloaded in *FASTQ* file format from Sequence Read Archive (SRA) database [87] (SRA Accession: SRP011546) with SRA Tool Kit v.2.9.0 using the following command: *fastq-dump --gzip --skip-technical --readids --dumpbase --clip --split-3*. Single cell DNA methylome sequencing data from another published study [76] were downloaded in BED file format from Gene Expression Omnibus (GEO Accession: GSE81233) with *wge*t command line utility of the Linux environment.

### Read mapping and expression profiling of genes and repeats

Human reference genome hg19 and its corresponding gene annotation in *GTF* format were downloaded from the UCSC Genome Browser (https://genome.ucsc.edu/). The *rsem-prepare-reference* command of RSEM tool v.1.3.0 [88] was used to build the Bowtie-2 [89] indices. For the alignment of RNA-seq reads to reference transcriptome and the measurement of relative abundances at gene level, we employed the *rsem-calculate-expression* script of RSEM with default parameters. We made use of Bowtie v.2–2.3.4 in both creating the indices and alignment step. We filtered out the genes with an expression level < 1 Transcripts Per Million (TPM) in all developmental stages and these genes were not considered for downstream analysis. For the estimation of repeat expressions at genome-wide level, we implemented a previously developed analysis pipeline; RepEnrich2 (https://github.com/nerettilab/RepEnrich2). The details of this computational approach can be found where it was initially described [27]. Estimated count values per repeat for each sample were normalised against the library size of a given sample and then Counts Per Million (CPM) values were calculated across all samples. Only the repeats having expression ≥ 1 CPM in all single cells of at least one particular developmental stage were included in downstream analysis in order to reduce noise in the expression matrix.

To determine the significance of differences in repeat expression across the developmental stages, we used the edgeR package v3.24.3 [90] of R statistical computation environment v3.4.4 (http://www.R-project.org) and compared the expression level at a given stage with the preceding one. Trimmed Mean of M-values (TMM) normalisation was applied to the count values from Repenrich2 and a generalised linear model was set up with developmental stage as factor. Then, dispersion estimation was done with estimateDisp function, and appropriate contrast statistics were employed using glmFit function of edgeR.

### Weighted Gene co-expression network (WGCNA) analysis

To identify co-expressed gene modules of the developing human embryos, we employed Weighted Gene Co-expression Network (WGCNA) analysis [67] in R environment and TPM values of coding genes were used as input. A signed weighted correlation network was built by calculating correlation coefficient value between all gene pairs across the developmental stages from 124 single cells individually. Then, the soft threshold value of the correlation matrix was set to 12, which is the default value for WGCNA analysis, and the adjacency matrix was created. In order to group together the genes showing high similarity in their expression pattern over time, we used average linkage hierarchical clustering method and co-expression network modules were determined with the Dynamic Tree Cut Algorithm [91]. In this step, we set minimum module size to 30 genes. Expression profile of each network module was summarised by calculating the module eigengene individually, representing the first principal component of the corresponding module. In the last step of the network analysis, the modules with highly correlated eigengenes (r ≥ 0.7) were merged into a single module.

### Enrichment analysis of repeats

For each gene, repeat elements located within the 2 kb upstream region of the transcription start site (TSS) were determined in order to discover the potential contribution of repeats to nearby gene regulation. We utilised hg19 reference genome and RepeatMasker annotation that were downloaded from the UCSC database, and overlapping genomic features were detected with the *intersectBed* command of bedtools [92]. We calculated ES of repeat elements for each network as follows:

$$ {\mathrm{ES}}_{\mathrm{X}}=\left(\mathrm{r}/\mathrm{R}\right)/\left(\mathrm{g}/\mathrm{G}\right) $$

- where R is the total number of all repeats located within the 2 kb upstream regions of the module genes,
- G is the total number of all repeats located within the 2 kb upstream regions of all genes in the genome,
- r is the total number of repeat of interest located within the 2 kb upstream regions of the module genes,
- g is the total number of repeat of interest located within the 2 kb upstream regions of all genes in the genome.

The statistical significance of each enrichment score was calculated with the Fisher’s exact test as we previously described in one of our studies [93]. Any repeat element was considered as significantly enriched and included in further analysis if it was associated with at least 5 genes within each module, with ES ≥ 1.5 and *p*-value ≤0.0001.

### Determining the methylation levels of repeat regions

Methylation levels of repeat regions were calculated with an in-house R script. Here, we first identified CpG sites falling within each repeat coordinates collected from the UCSC database. CpG percentages were calculated by taking the ratio of the number of reads supporting methylated to that of total reads. A similar approach was also used by Zhu et al. in the estimation of DNA methylation levels of repeat regions in human pre-implantation embryos [76]. We utilised a stringent criterion to call DNA methylation levels and removed CpGs with a coverage of less than 5 reads as it was done previously by Stadler et al. [94]. In other words, only the CpGs that were covered at least 5× were included in the quantification of DNA methylation levels, which were identified by taking the ratio of the number of reads supporting methylated to that of total reads (methylated and unmethylated) for each repeat element. The R script for our methylation analysis could be found in the Additional file 10.

### Statistical analysis and graphical representation

All statistical analyses in this study were conducted within the R computation environment. Pearson’s correlation and enrichment score analyses were performed with *cor.test* and *fisher.test* functions, respectively. Heat-maps were drawn with *heatmap.2* function of R and all expression values were scaled by row.

Z-scores were calculated as the following:

$$ \mathrm{z}-\mathrm{score}=\left(\mathrm{average}\ \mathrm{expression}\ \mathrm{of}\ \mathrm{the}\ \mathrm{given}\ \mathrm{repeat}\ \mathrm{in}\ \mathrm{the}\ \mathrm{specified}\ \mathrm{developmental}\ \mathrm{stage}-\mathrm{mean}\ \mathrm{expression}\ \mathrm{for}\ \mathrm{the}\ \mathrm{average}\ \mathrm{expression}\mathrm{s}\ \mathrm{for}\ \mathrm{the}\ \mathrm{given}\ \mathrm{repeat}\ \mathrm{across}\ \mathrm{all}\ \mathrm{developmental}\ \mathrm{stage}\mathrm{s}\right)/\left(\mathrm{standard}\ \mathrm{deviation}\ \mathrm{of}\ \mathrm{the}\ \mathrm{average}\ \mathrm{expression}\ \mathrm{for}\ \mathrm{the}\ \mathrm{given}\ \mathrm{repeat}\ \mathrm{across}\ \mathrm{all}\ \mathrm{developmental}\ \mathrm{stage}\mathrm{s}\right) $$

For clustering, Euclidian method was utilised and PCA was conducted with *plotPCA* function. For the visualisation of expression and methylome data, we made use of ggplot2 package. One-way ANOVA followed by Tukey’s multiple comparison tests was performed to compare the differences of CVs and methylation percentages between different developmental stages. Those with *p*-adj < 0.001 were considered to be statistically significant.

## Additional files

Additional file 1: **Figure S1.** Principal component (PCA) analysis of gene expression. Each circle represents a single cell in the corresponding developmental stage. **Figure S2.** Box-plot representations of repeat and gene expressions obtained from single cells across stages of human pre-implantation and different passages (P0 and P10) of embryonic stem cells (ESC). **a** Expression levels of 1116 repeat elements. **b** Expression levels of UCSC annotated genes. **Figure S3.** Box-plot representations of expression levels of all members of each major repeat class across stages of human pre-implantation and different passages (P0 and P10) of embryonic stem cells (ESC). **Figure S4.** Line plots representing the expression levels of each member of satellite repeat family. Error bars indicate standard errors of the mean. Repeats that are expressed below the 1-CPM threshold are not shown. **Figure S5.** Heat-map representing the expression levels of genes in stage specific modules as analysed with weighted gene co-expression network analysis (WGCNA). Gene Ontology terms enriched in these modules and their corresponding *p*-values are listed next to them. **Figure S6.** Line plots representing mean expression levels of repeats that exhibit higher than 3.5 fold enrichment in any WGCNA module. In addition to 11 repeats which are above the 3.5 threshold; FLAM_C, SVA_D and SVA_F levels are also shown. Error bars indicate the standard error of the mean. **Figure S7.** Scatter plots representing the correlation between developmentally important genes and the relevant repeats. **Figure S8.** Additional module-repeat correlations for repeats that are below the 3.5 enrichment threshold. **Figure S9.** Line plots representing mean DNA methylation levels of different members of satellite repeat family. Error bars indicate the standard error of the mean (PDF 18534 kb)

Additional file 2: **Table S1.** Number of embryos and single cells used in the RNA-seq analysis (XLSX 39 kb)

Additional file 3: **Table S2.** CPM values for repeats (XLSX 1675 kb)

Additional file 4: **Table S3.** Fraction counts of repeats by RepEnrich2 (XLSX 782 kb)

Additional file 5: **Table S4.** Repeat expression fold changes and FDR values (XLSX 266 kb)

Additional file 6: **Table S5.** TPM values for genes by RSEM (XLSX 17108 kb)

Additional file 7: **Table S6.** Expected counts for genes (XLSX 16383 kb)

Additional file 8: **Table S7.** List of WGCNA module specific genes (XLSX 272 kb)

Additional file 9: **Table S8.** Number of single cells used for DNA-methylation sequencing data and their GEO accession numbers (XLSX 34 kb)

Additional file 10: R script for methylation percentage analysis (R 4 kb)

Additional file 11: **Table S9.** Methylation percentages of repeats (XLSX 2183 kb)

Additional file 12: **Table S10.** Statistical analysis of DNA methylation percentages for repeats (ANOVA followed by Tukey test results) (XLSX 66 kb)

## Abbreviations

5caC: 5-carboxylcytosine
5fC: 5-formylcytosine
5hmC: 5-hydroxymethylcytosine
5mC: 5-methylcytosine
CPM: Counts Per Million
CV: Coefficient of variation
ES: Enrichment score
ESC: Embryonic stem cell
FDR: False discovery rate
GEO: Gene expression omnibus
ICM: Inner cell mass
LINE: Long interspersed nuclear element
LTR: Long terminal repeat
NLB: Nucleolar like body
P0: Passage 0
P10: Passage 10
PCA: Principal component analysis
RNA-seq: RNA sequencing
SINE: Short interspersed nuclear element
SRA: Sequence read archive
SVA: SINE-VNTR-Alu
TPM: Transcripts Per Million
TSS: Transcription start site
WGCNA: Weighted gene co-expression network analysis

## Authors’ information

CY was a full-time faculty member at Izmir University of Economics and also an honorary research associate at Izmir Biomedicine and Genome Center as well as Imperial College London while this study was being performed.

## References

[1] Cantone I, Fisher AG (2013). Epigenetic programming and reprogramming during development. Nat Struct Mol Biol.

[2] Fraser R, Lin CJ (2016). Epigenetic reprogramming of the zygote in mice and men: on your marks, get set, go!. Reproduction.

[3] Nashun B, Hill PW, Hajkova P (2015). Reprogramming of cell fate: epigenetic memory and the erasure of memories past. EMBO J.

[4] Saksouk N, Simboeck E, Dejardin J (2015). Constitutive heterochromatin formation and transcription in mammals. Epigenetics Chromatin.

[5] Vos LJ, Famulski JK, Chan GK (2006). How to build a centromere: from centromeric and pericentromeric chromatin to kinetochore assembly. Biochem Cell Biol.

[6] Daujat S, Weiss T, Mohn F, Lange UC, Ziegler-Birling C, Zeissler U, Lappe M, Schubeler D, Torres-Padilla ME, Schneider R (2009). H3K64 trimethylation marks heterochromatin and is dynamically remodeled during developmental reprogramming. Nat Struct Mol Biol.

[7] Kourmouli N, Jeppesen P, Mahadevhaiah S, Burgoyne P, Wu R, Gilbert DM, Bongiorni S, Prantera G, Fanti L, Pimpinelli S (2004). Heterochromatin and tri-methylated lysine 20 of histone H4 in animals. J Cell Sci.

[8] Liu H, Kim JM, Aoki F (2004). Regulation of histone H3 lysine 9 methylation in oocytes and early pre-implantation embryos. Development.

[9] Puschendorf M, Terranova R, Boutsma E, Mao X, Isono K, Brykczynska U, Kolb C, Otte AP, Koseki H, Orkin SH (2008). PRC1 and Suv39h specify parental asymmetry at constitutive heterochromatin in early mouse embryos. Nat Genet.

[10] Ahmed K, Dehghani H, Rugg-Gunn P, Fussner E, Rossant J, Bazett-Jones DP (2010). Global chromatin architecture reflects pluripotency and lineage commitment in the early mouse embryo. PLoS One.

[11] Aguirre-Lavin T, Adenot P, Bonnet-Garnier A, Lehmann G, Fleurot R, Boulesteix C, Debey P, Beaujean N (2012). 3D-FISH analysis of embryonic nuclei in mouse highlights several abrupt changes of nuclear organization during preimplantation development. BMC Dev Biol.

[12] Burns KH, Viveiros MM, Ren Y, Wang P, DeMayo FJ, Frail DE, Eppig JJ, Matzuk MM (2003). Roles of NPM2 in chromatin and nucleolar organization in oocytes and embryos. Science.

[13] Burton A, Torres-Padilla ME (2014). Chromatin dynamics in the regulation of cell fate allocation during early embryogenesis. Nat Rev Mol Cell Biol.

[14] Probst AV, Santos F, Reik W, Almouzni G, Dean W (2007). Structural differences in centromeric heterochromatin are spatially reconciled on fertilisation in the mouse zygote. Chromosoma.

[15] Fadloun A, Eid A, Torres-Padilla ME (2013). Mechanisms and dynamics of heterochromatin formation during mammalian development: closed paths and open questions. Curr Top Dev Biol.

[16] Martin C, Beaujean N, Brochard V, Audouard C, Zink D, Debey P (2006). Genome restructuring in mouse embryos during reprogramming and early development. Dev Biol.

[17] van de Werken C, van der Heijden GW, Eleveld C, Teeuwssen M, Albert M, Baarends WM, Laven JS, Peters AH, Baart EB (2014). Paternal heterochromatin formation in human embryos is H3K9/HP1 directed and primed by sperm-derived histone modifications. Nat Commun.

[18] Jachowicz JW, Santenard A, Bender A, Muller J, Torres-Padilla ME (2013). Heterochromatin establishment at pericentromeres depends on nuclear position. Genes Dev.

[19] Probst AV, Okamoto I, Casanova M, El Marjou F, Le Baccon P, Almouzni G (2010). A strand-specific burst in transcription of pericentric satellites is required for chromocenter formation and early mouse development. Dev Cell.

[20] Jagannathan M, Yamashita YM (2018). Function of junk: Pericentromeric Satellite DNA in chromosome maintenance. Cold Spring Harbor symposia on quantitative biology.

[21] Casanova M, Pasternak M, El Marjou F, Le Baccon P, Probst AV, Almouzni G (2013). Heterochromatin reorganization during early mouse development requires a single-stranded noncoding transcript. Cell Rep.

[22] Magaraki A, van der Heijden G, Sleddens-Linkels E, Magarakis L, van Cappellen WA, Peters A, Gribnau J, Baarends WM, Eijpe M (2017). Silencing markers are retained on pericentric heterochromatin during murine primordial germ cell development. Epigenetics Chromatin.

[23] Maison C, Bailly D, Roche D, Montes de Oca R, Probst AV, Vassias I, Dingli F, Lombard B, Loew D, Quivy JP (2011). SUMOylation promotes de novo targeting of HP1alpha to pericentric heterochromatin. Nat Genet.

[24] Maison C, Quivy JP, Almouzni G (2016). Suv39h1 links the SUMO pathway to constitutive heterochromatin. Mol Cell Oncol.

[25] de Koning AP, Gu W, Castoe TA, Batzer MA, Pollock DD (2011). Repetitive elements may comprise over two-thirds of the human genome. PLoS Genet.

[26] Lander ES, Linton LM, Birren B, Nusbaum C, Zody MC, Baldwin J, Devon K, Dewar K, Doyle M, FitzHugh W (2001). Initial sequencing and analysis of the human genome. Nature.

[27] Criscione SW, Zhang Y, Thompson W, Sedivy JM, Neretti N (2014). Transcriptional landscape of repetitive elements in normal and cancer human cells. BMC Genomics.

[28] Ting DT, Lipson D, Paul S, Brannigan BW, Akhavanfard S, Coffman EJ, Contino G, Deshpande V, Iafrate AJ, Letovsky S (2011). Aberrant overexpression of satellite repeats in pancreatic and other epithelial cancers. Science.

[29] Zhu Q, Pao GM, Huynh AM, Suh H, Tonnu N, Nederlof PM, Gage FH, Verma IM (2011). BRCA1 tumour suppression occurs via heterochromatin-mediated silencing. Nature.

[30] Lee E, Iskow R, Yang L, Gokcumen O, Haseley P, Luquette LJ, Lohr JG, Harris CC, Ding L, Wilson RK (2012). Landscape of somatic retrotransposition in human cancers. Science.

[31] Crow MK (2010). Long interspersed nuclear elements (LINE-1): potential triggers of systemic autoimmune disease. Autoimmunity.

[32] Hancks DC, Kazazian HH (2016). Roles for retrotransposon insertions in human disease. Mob DNA.

[33] Gao L, Wu K, Liu Z, Yao X, Yuan S, Tao W, Yi L, Yu G, Hou Z, Fan D (2018). Chromatin accessibility landscape in human early embryos and its association with evolution. Cell.

[34] Grow EJ, Flynn RA, Chavez SL, Bayless NL, Wossidlo M, Wesche DJ, Martin L, Ware CB, Blish CA, Chang HY (2015). Intrinsic retroviral reactivation in human preimplantation embryos and pluripotent cells. Nature.

[35] Boroviak Thorsten, Stirparo Giuliano G., Dietmann Sabine, Hernando-Herraez Irene, Mohammed Hisham, Reik Wolf, Smith Austin, Sasaki Erika, Nichols Jennifer, Bertone Paul (2018). Single cell transcriptome analysis of human, marmoset and mouse embryos reveals common and divergent features of preimplantation development. Development.

[36] Yan L, Yang M, Guo H, Yang L, Wu J, Li R, Liu P, Lian Y, Zheng X, Yan J (2013). Single-cell RNA-Seq profiling of human preimplantation embryos and embryonic stem cells. Nat Struct Mol Biol.

[37] Guo H, Zhu P, Yan L, Li R, Hu B, Lian Y, Yan J, Ren X, Lin S, Li J (2014). The DNA methylation landscape of human early embryos. Nature.

[38] Li Lin, Guo Fan, Gao Yun, Ren Yixin, Yuan Peng, Yan Liying, Li Rong, Lian Ying, Li Jingyun, Hu Boqiang, Gao Junpeng, Wen Lu, Tang Fuchou, Qiao Jie (2018). Single-cell multi-omics sequencing of human early embryos. Nature Cell Biology.

[39] Petropoulos S, Edsgard D, Reinius B, Deng Q, Panula SP, Codeluppi S, Plaza Reyes A, Linnarsson S, Sandberg R, Lanner F (2016). Single-cell RNA-Seq reveals lineage and X chromosome dynamics in human preimplantation embryos. Cell.

[40] Wu J, Xu J, Liu B, Yao G, Wang P, Lin Z, Huang B, Wang X, Li T, Shi S (2018). Chromatin analysis in human early development reveals epigenetic transition during ZGA. Nature.

[41] Bao W, Kojima KK, Kohany O (2015). Repbase update, a database of repetitive elements in eukaryotic genomes. Mob DNA.

[42] Xue Z, Huang K, Cai C, Cai L, Jiang CY, Feng Y, Liu Z, Zeng Q, Cheng L, Sun YE (2013). Genetic programs in human and mouse early embryos revealed by single-cell RNA sequencing. Nature.

[43] Braude P, Bolton V, Moore S (1988). Human gene expression first occurs between the four- and eight-cell stages of preimplantation development. Nature.

[44] Jukam D, Shariati SAM, Skotheim JM (2017). Zygotic genome activation in vertebrates. Dev Cell.

[45] Niakan KK, Han J, Pedersen RA, Simon C, Pera RA (2012). Human pre-implantation embryo development. Development.

[46] Blakeley P, Fogarty NM, del Valle I, Wamaitha SE, Hu TX, Elder K, Snell P, Christie L, Robson P, Niakan KK (2015). Defining the three cell lineages of the human blastocyst by single-cell RNA-seq. Development.

[47] Chazaud C, Yamanaka Y (2016). Lineage specification in the mouse preimplantation embryo. Development.

[48] De Paepe C, Krivega M, Cauffman G, Geens M, Van de Velde H (2014). Totipotency and lineage segregation in the human embryo. Mol Hum Reprod.

[49] Petropoulos S, Edsgard D, Reinius B, Deng Q, Panula SP, Codeluppi S, Reyes AP, Linnarsson S, Sandberg R, Lanner F (2016). Single-cell RNA-Seq reveals lineage and X chromosome dynamics in human preimplantation embryos. Cell.

[50] Piotrowska-Nitsche K, Zernicka-Goetz M (2005). Spatial arrangement of individual 4-cell stage blastomeres and the order in which they are generated correlate with blastocyst pattern in the mouse embryo. Mech Dev.

[51] Torres-Padilla ME, Parfitt DE, Kouzarides T, Zernicka-Goetz M (2007). Histone arginine methylation regulates pluripotency in the early mouse embryo. Nature.

[52] Mantsoki A, Devailly G, Joshi A (2016). Gene expression variability in mammalian embryonic stem cells using single cell RNA-seq data. Comput Biol Chem.

[53] Garrido-Ramos Manuel (2017). Satellite DNA: An Evolving Topic. Genes.

[54] Mayor R, Izquierdo-Bouldstridge A, Millan-Arino L, Bustillos A, Sampaio C, Luque N, Jordan A (2015). Genome distribution of replication-independent histone H1 variants shows H1.0 associated with nucleolar domains and H1X associated with RNA polymerase II-enriched regions. J Biol Chem.

[55] Floutsakou I, Agrawal S, Nguyen TT, Seoighe C, Ganley AR, McStay B (2013). The shared genomic architecture of human nucleolar organizer regions. Genome Res.

[56] Bowden DW, Krawchuk MD, Weaver EJ, Howard TD, Knowlton RG, Rao PN, Pettenati MJ, Hayworth R, Wagner BJ, Rothschild CB (1995). D20S16 is a complex interspersed repeated sequence: genetic and physical analysis of the locus. Genomics.

[57] Ge SX (2017). Exploratory bioinformatics investigation reveals importance of "junk" DNA in early embryo development. BMC Genomics.

[58] Lu F, Liu Y, Inoue A, Suzuki T, Zhao K, Zhang Y (2016). Establishing chromatin regulatory landscape during mouse preimplantation development. Cell.

[59] Smith ZD, Chan MM, Humm KC, Karnik R, Mekhoubad S, Regev A, Eggan K, Meissner A (2014). DNA methylation dynamics of the human preimplantation embryo. Nature.

[60] Wang C, Liu X, Gao Y, Yang L, Li C, Liu W, Chen C, Kou X, Zhao Y, Chen J (2018). Reprogramming of H3K9me3-dependent heterochromatin during mammalian embryo development. Nat Cell Biol.

[61] Cheng Y, Lund E, Kahan BW, Dahlberg JE (1997). Control of mouse U1 snRNA gene expression during in vitro differentiation of mouse embryonic stem cells. Nucleic Acids Res.

[62] Lobo SM, Marzluff WF, Seufert AC, Dean WL, Schultz GA, Simerly C, Schatten G (1988). Localization and expression of U1 RNA in early mouse embryo development. Dev Biol.

[63] Lund E, Kahan B, Dahlberg JE (1985). Differential control of U1 small nuclear RNA expression during mouse development. Science.

[64] Enright CA, Maxwell ES, Eliceiri GL, Sollner-Webb B (1996). 5′ETS rRNA processing facilitated by four small RNAs: U14, E3, U17, and U3. RNA.

[65] Peculis BA (1997). The sequence of the 5′ end of the U8 small nucleolar RNA is critical for 5.8S and 28S rRNA maturation. Mol Cell Biol.

[66] Tiedge H, Chen W, Brosius J (1993). Primary structure, neural-specific expression, and dendritic location of human BC200 RNA. J Neurosci.

[67] Langfelder P, Horvath S (2008). WGCNA: an R package for weighted correlation network analysis. BMC Bioinf.

[68] Zhang B, Horvath S (2005). A general framework for weighted gene co-expression network analysis. Stat Appl Genet Mol Biol.

[69] The Gene Ontology C (2017). Expansion of the Gene Ontology knowledgebase and resources. Nucleic Acids Res.

[70] Yagi M, Uchiumi T, Takazaki S, Okuno B, Nomura M, Yoshida S, Kanki T, Kang D (2012). p32/gC1qR is indispensable for fetal development and mitochondrial translation: importance of its RNA-binding ability. Nucleic Acids Res.

[71] Dunwell Thomas L., Holland Peter W. H. (2017). A sister of NANOG regulates genes expressed in pre-implantation human development. Open Biology.

[72] Lee A, Jimenez A, Cui G, Haeseleer F (2007). Phosphorylation of the Ca2+−binding protein CaBP4 by protein kinase C zeta in photoreceptors. J Neurosci.

[73] Marcho C, Cui W, Mager J (2015). Epigenetic dynamics during preimplantation development. Reproduction.

[74] Okae H, Chiba H, Hiura H, Hamada H, Sato A, Utsunomiya T, Kikuchi H, Yoshida H, Tanaka A, Suyama M (2014). Genome-wide analysis of DNA methylation dynamics during early human development. PLoS Genet.

[75] Saitou M, Kagiwada S, Kurimoto K (2012). Epigenetic reprogramming in mouse pre-implantation development and primordial germ cells. Development.

[76] Zhu P, Guo H, Ren Y, Hou Y, Dong J, Li R, Lian Y, Fan X, Hu B, Gao Y (2018). Single-cell DNA methylome sequencing of human preimplantation embryos. Nat Genet.

[77] Yang J, Liu P (2017). Cell lineage specification at single cell resolution. Stem Cell Investig.

[78] Biase FH, Cao X, Zhong S (2014). Cell fate inclination within 2-cell and 4-cell mouse embryos revealed by single-cell RNA sequencing. Genome Res.

[79] Li L, Zheng P, Dean J (2010). Maternal control of early mouse development. Development.

[80] Martens JH, O'Sullivan RJ, Braunschweig U, Opravil S, Radolf M, Steinlein P, Jenuwein T (2005). The profile of repeat-associated histone lysine methylation states in the mouse epigenome. EMBO J.

[81] Natisvili T, Yandim C, Silva R, Emanuelli G, Krueger F, Nageshwaran S, Festenstein R (2016). Transcriptional activation of Pericentromeric Satellite repeats and disruption of Centromeric clustering upon proteasome inhibition. PLoS One.

[82] Lee MT, Bonneau AR, Giraldez AJ (2014). Zygotic genome activation during the maternal-to-zygotic transition. Annu Rev Cell Dev Biol.

[83] Solovyov A, Vabret N, Arora KS, Snyder A, Funt SA, Bajorin DF, Rosenberg JE, Bhardwaj N, Ting DT, Greenbaum BD (2018). Global Cancer transcriptome quantifies repeat element polarization between immunotherapy responsive and T cell suppressive classes. Cell Rep.

[84] Shirai A, Kawaguchi T, Shimojo H, Muramatsu D, Ishida-Yonetani M, Nishimura Y, Kimura H, Nakayama JI, Shinkai Y. Impact of nucleic acid and methylated H3K9 binding activities of Suv39h1 on its heterochromatin assembly. eLife. 2017;6. 10.7554/eLife.25317. 10.7554/eLife.25317 PMC5538823 28760201

[85] Papin C, Ibrahim A, Gras SL, Velt A, Stoll I, Jost B, Menoni H, Bronner C, Dimitrov S, Hamiche A (2017). Combinatorial DNA methylation codes at repetitive elements. Genome Res.

[86] Clark SJ, Argelaguet R, Kapourani CA, Stubbs TM, Lee HJ, Alda-Catalinas C, Krueger F, Sanguinetti G, Kelsey G, Marioni JC (2018). scNMT-seq enables joint profiling of chromatin accessibility DNA methylation and transcription in single cells. Nat Commun.

[87] Leinonen R, Sugawara H, Shumway M (2011). International nucleotide sequence database C: The sequence read archive. Nucleic Acids Res.

[88] Li B, Dewey CN (2011). RSEM: accurate transcript quantification from RNA-Seq data with or without a reference genome. BMC Bioinf.

[89] Langmead B, Salzberg SL (2012). Fast gapped-read alignment with bowtie 2. Nat Methods.

[90] Robinson MD, McCarthy DJ, Smyth GK (2010). edgeR: a Bioconductor package for differential expression analysis of digital gene expression data. Bioinformatics.

[91] Langfelder P, Zhang B, Horvath S (2008). Defining clusters from a hierarchical cluster tree: the dynamic tree cut package for R. Bioinformatics.

[92] Quinlan AR (2014). BEDTools: The Swiss-Army tool for genome feature analysis. Curr Protoc Bioinformatics.

[93] Karakulah G, Suner A (2017). PlanTEnrichment: a tool for enrichment analysis of transposable elements in plants. Genomics.

[94] Stadler MB, Murr R, Burger L, Ivanek R, Lienert F, Scholer A, van Nimwegen E, Wirbelauer C, Oakeley EJ, Gaidatzis D (2011). DNA-binding factors shape the mouse methylome at distal regulatory regions. Nature.

