# Supplementary material for: Expression dynamics of repetitive DNA in early human embryonic development
Source: BMC Genomics. 2019 May 31;20:439. doi: 10.1186/s12864-019-5803-1 (PMC6545021; doi:10.1186/s12864-019-5803-1)
Supplement: Supplementary file 1 — Figure S1. Principal component (PCA) analysis of gene expression. Each circle represents a single cell in the corresponding developmental stage. Figure S2. Box-plot representations of repeat and gene expressions obtained from single cells across stages of human pre-implantation and different passages (P0 and P10) of embryonic stem cells (ESC). a Expression levels of 1116 repeat elements. b Expression levels of UCSC annotated genes. Figure S3. Box-plot representations of expression levels of all members of each major repeat class across stages of human pre-implantation and different passages (P0 and P10) of embryonic stem cells (ESC). Figure S4. Line plots representing the expression levels of each member of satellite repeat family. Error bars indicate standard errors of the mean. Repeats that are expressed below the 1-CPM threshold are not shown. Figure S5. Heat-map representing the expression levels of genes in stage specific modules as analysed with weighted gene co-expression network analysis (WGCNA). Gene Ontology terms enriched in these modules and their corresponding p-values are listed next to them. Figure S6. Line plots representing mean expression levels of repeats that exhibit higher than 3.5 fold enrichment in any WGCNA module. In addition to 11 repeats which are above the 3.5 threshold; FLAM_C, SVA_D and SVA_F levels are also shown. Error bars indicate the standard error of the mean. Figure S7. Scatter plots representing the correlation between developmentally important genes and the relevant repeats. Figure S8. Additional module-repeat correlations for repeats that are below the 3.5 enrichment threshold. Figure S9. Line plots representing mean DNA methylation levels of different members of satellite repeat family. Error bars indicate the standard error of the mean (PDF 18534 kb) [file 12864_2019_5803_MOESM1_ESM.pdf]

Expression dynamics of repetitive DNA in early human embryonic development

*Cihangir Yandim, Gokhan Karakulah**

**Corresponding Author*

gokhan.karakulah@ibg.edu.tr

SUPPLEMENTARY FIGURES

Figure S1

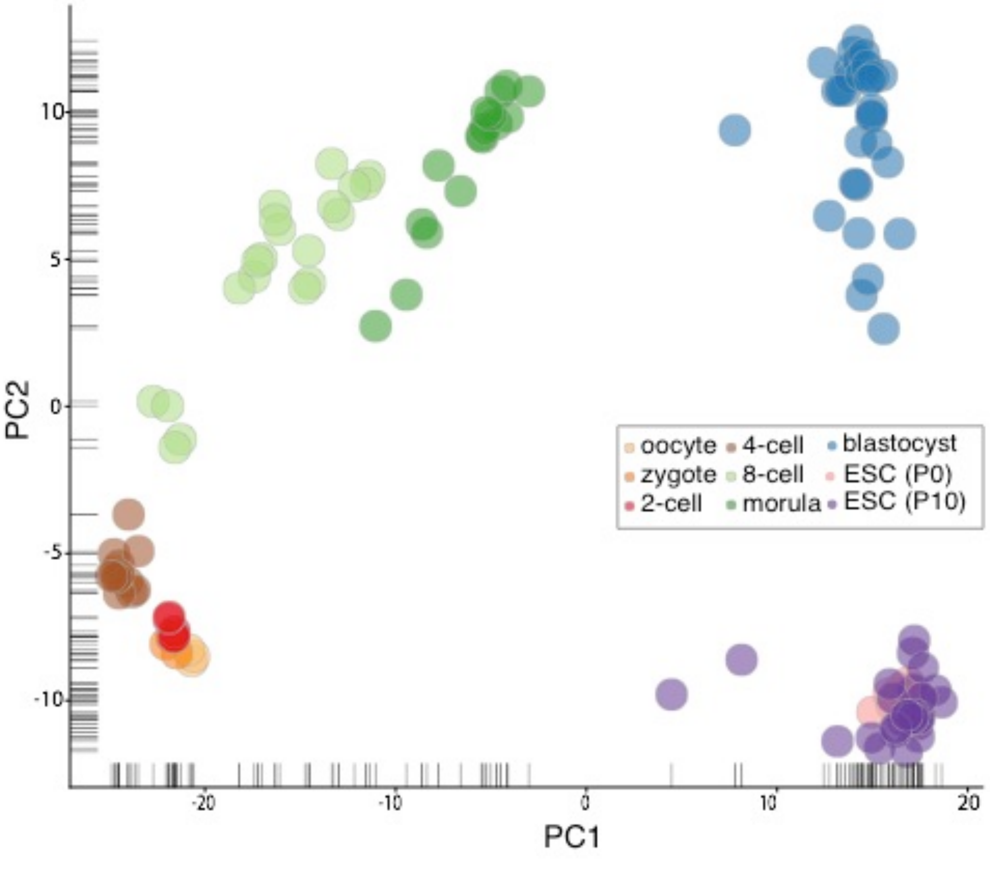

Figure S1. Principal component (PCA) analysis of gene expression. Each circle represents a single cell in the corresponding developmental stage.

Figure S2

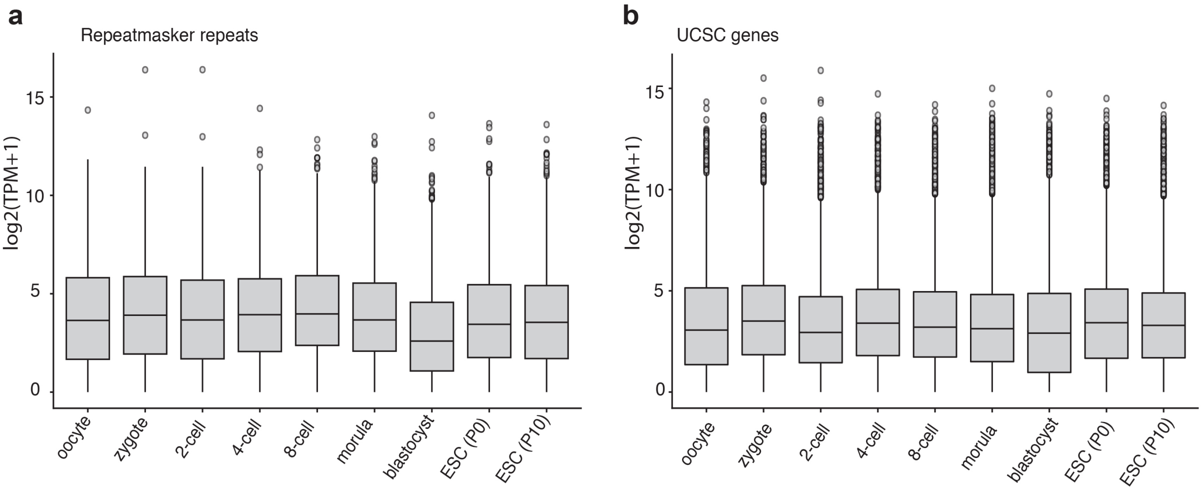

Figure S2. Box-plot representations of repeat and gene expressions obtained from single cells across stages of human pre-implantation and different passages (P0 and P10) of embryonic stem cells (ESC). **a** Expression levels of 1116 repeat elements. **b** Expression levels of UCSC annotated genes.

Figure S3

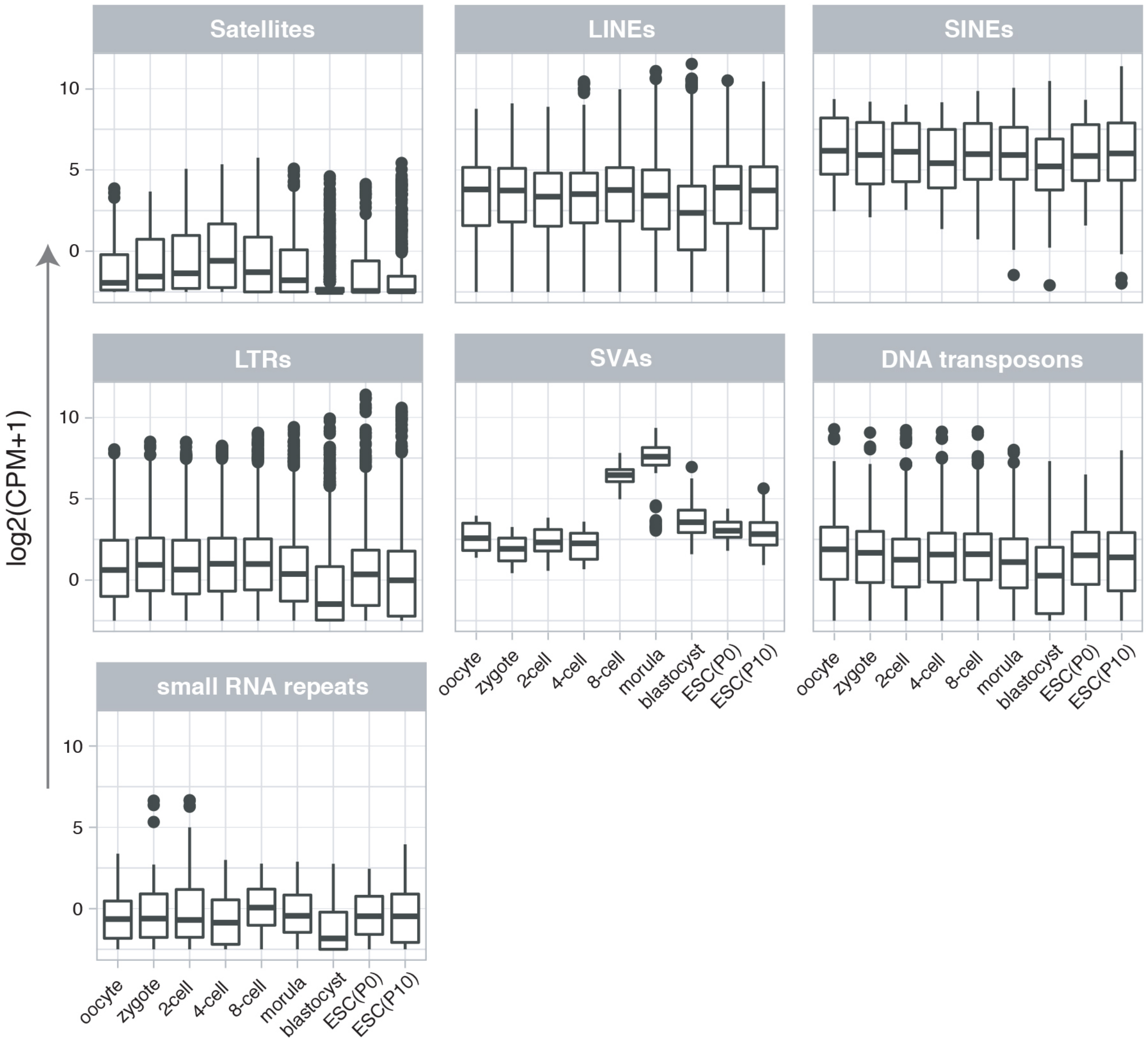

Figure S3. Box-plot representations of expression levels of all members of each major repeat class across stages of human pre-implantation and different passages (P0 and P10) of embryonic stem cells (ESC).

Figure S4

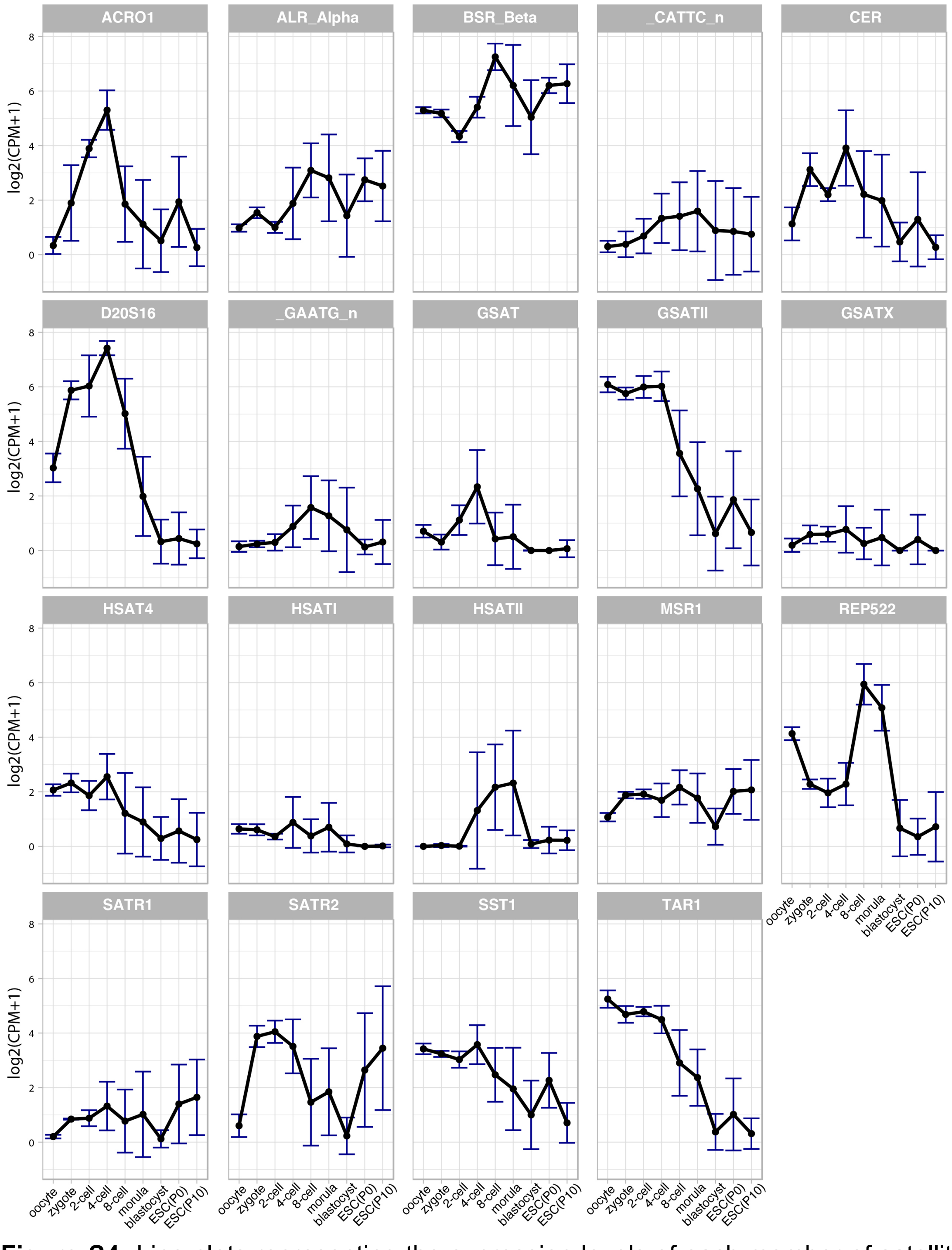

Figure S4. Line plots representing the expression levels of each member of satellite repeat family. Error bars indicate standard errors of the mean. Repeats that are expressed below the 1-CPM threshold are not shown.

Figure S5

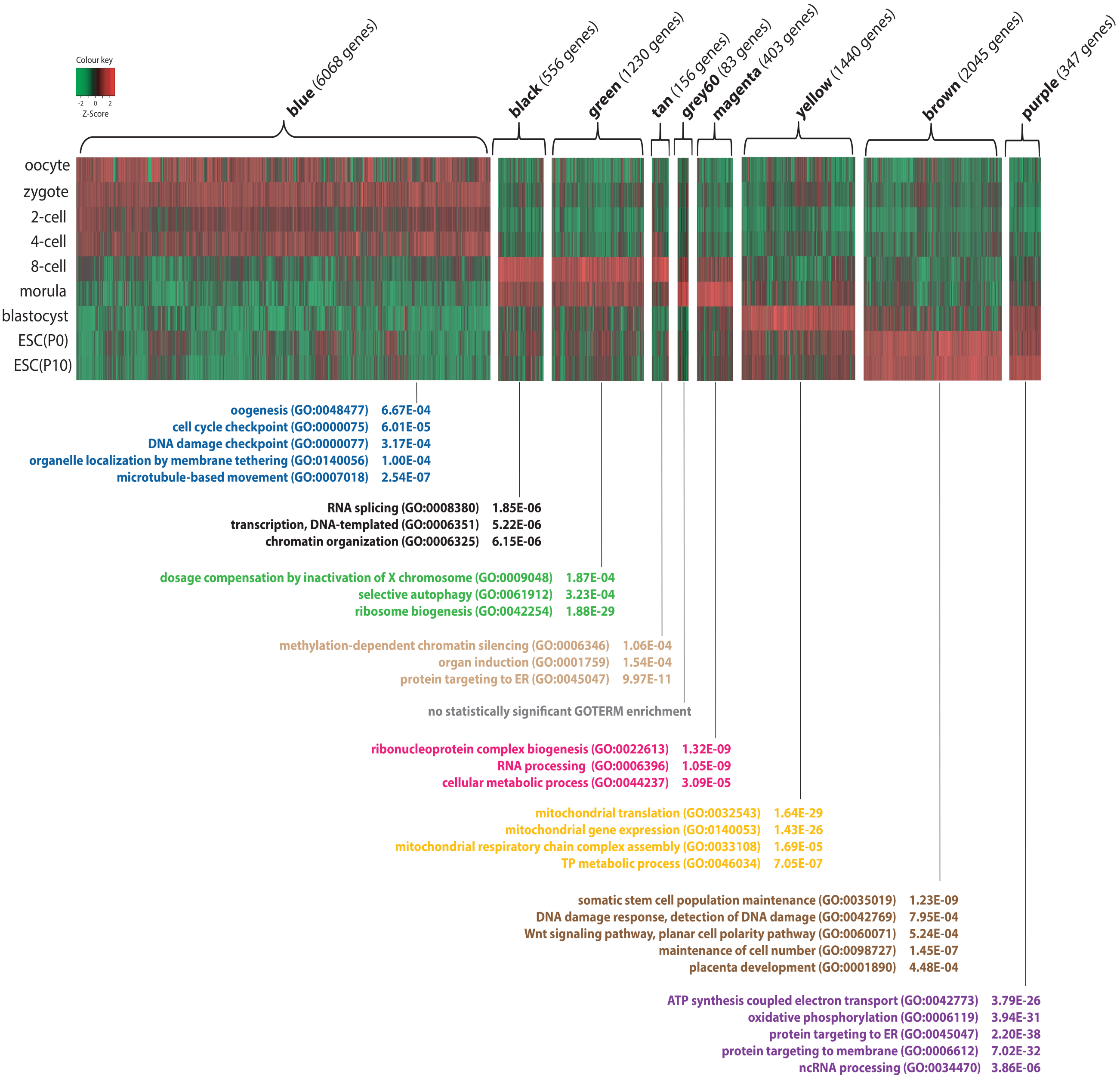

Figure S5. Heat-map representing the expression levels of genes in stage specific modules as analysed with weighted gene co-expression network analysis (WGCNA). GO terms enriched in these modules and their corresponding p-values are listed next to them.

Figure S6

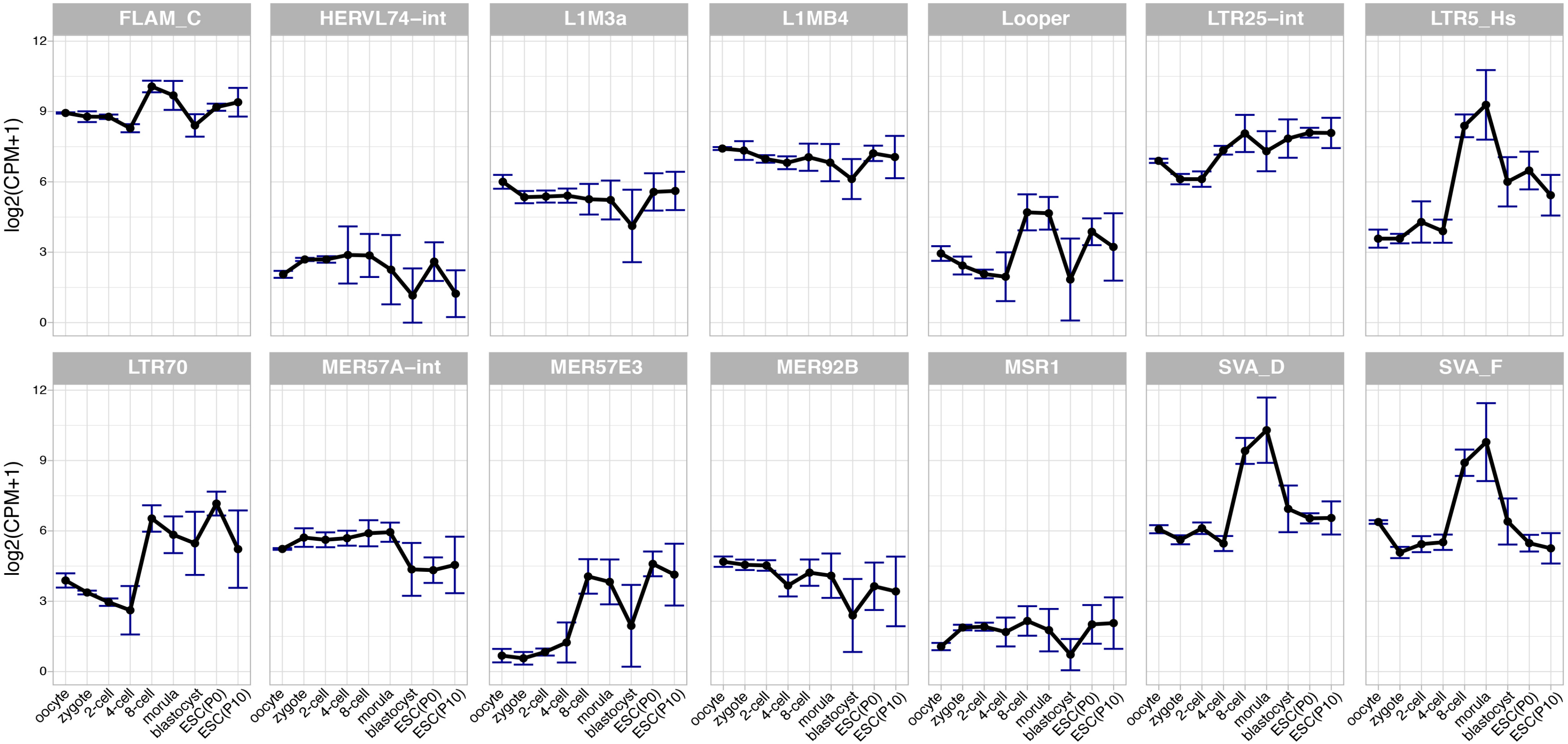

Figure S6. Line plots representing mean expression levels of repeats that exhibit higher than 3.5 fold enrichment in any WGCNA module. In addition to 11 repeats which are above the 3.5 threshold; FLAM_C, SVA_D and SVA_F levels are also shown. Error bars indicate the standard error of the mean.

Figure S7

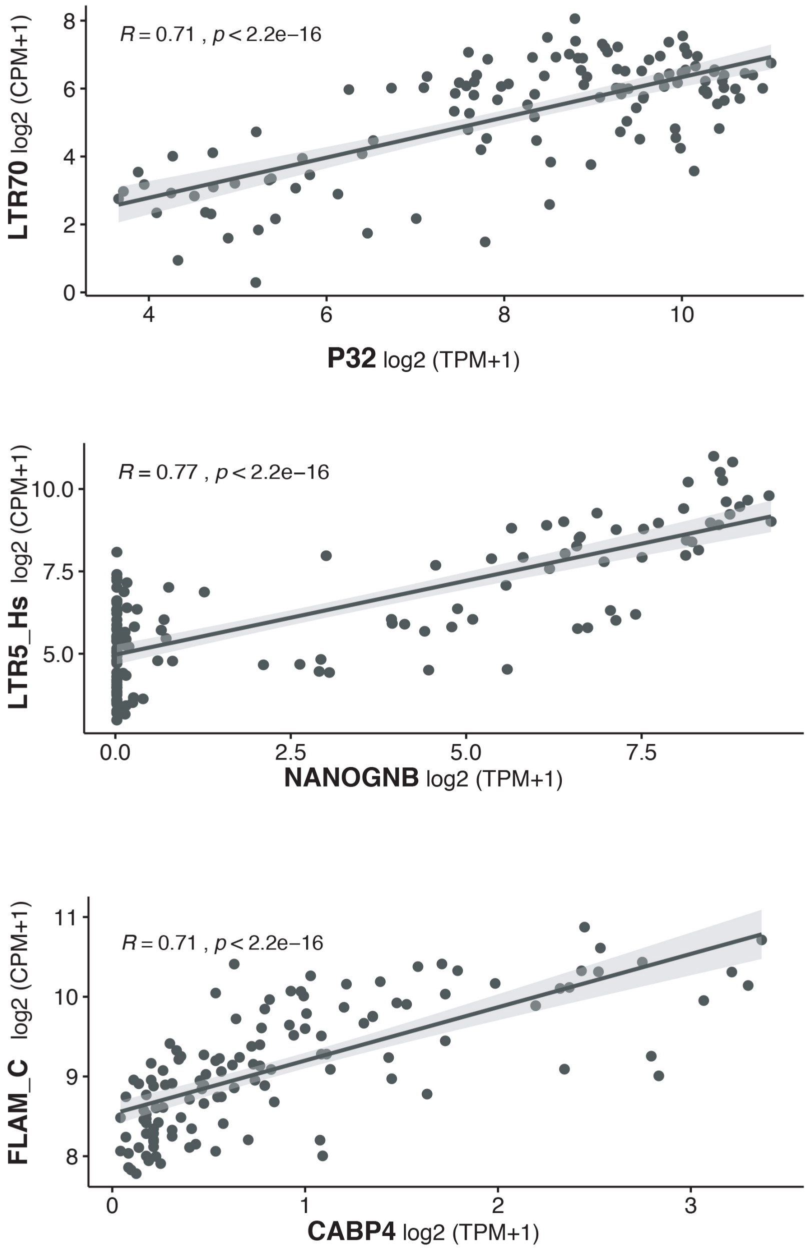

Figure S7. Scatter plots representing the correlation between developmentally vital genes and the relevant repeats.

Figure S8

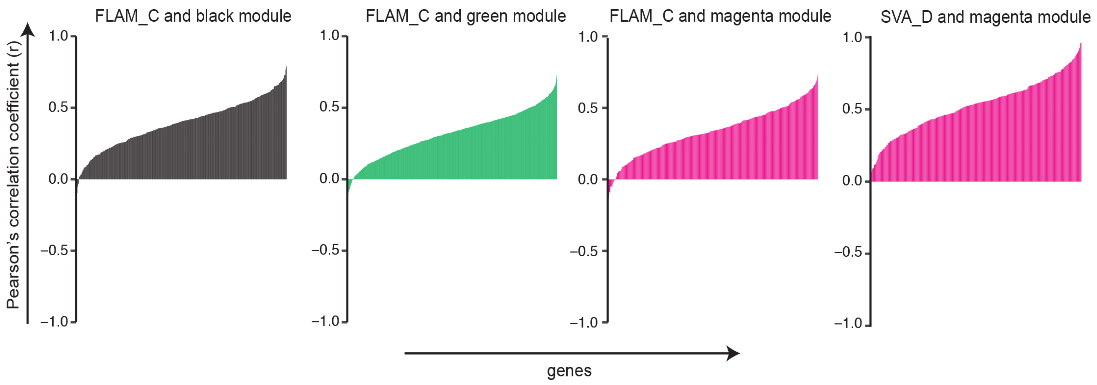

Figure S8. Additional module-repeat correlations for repeats that are below the 3.5 enrichment threshold.

Figure S9

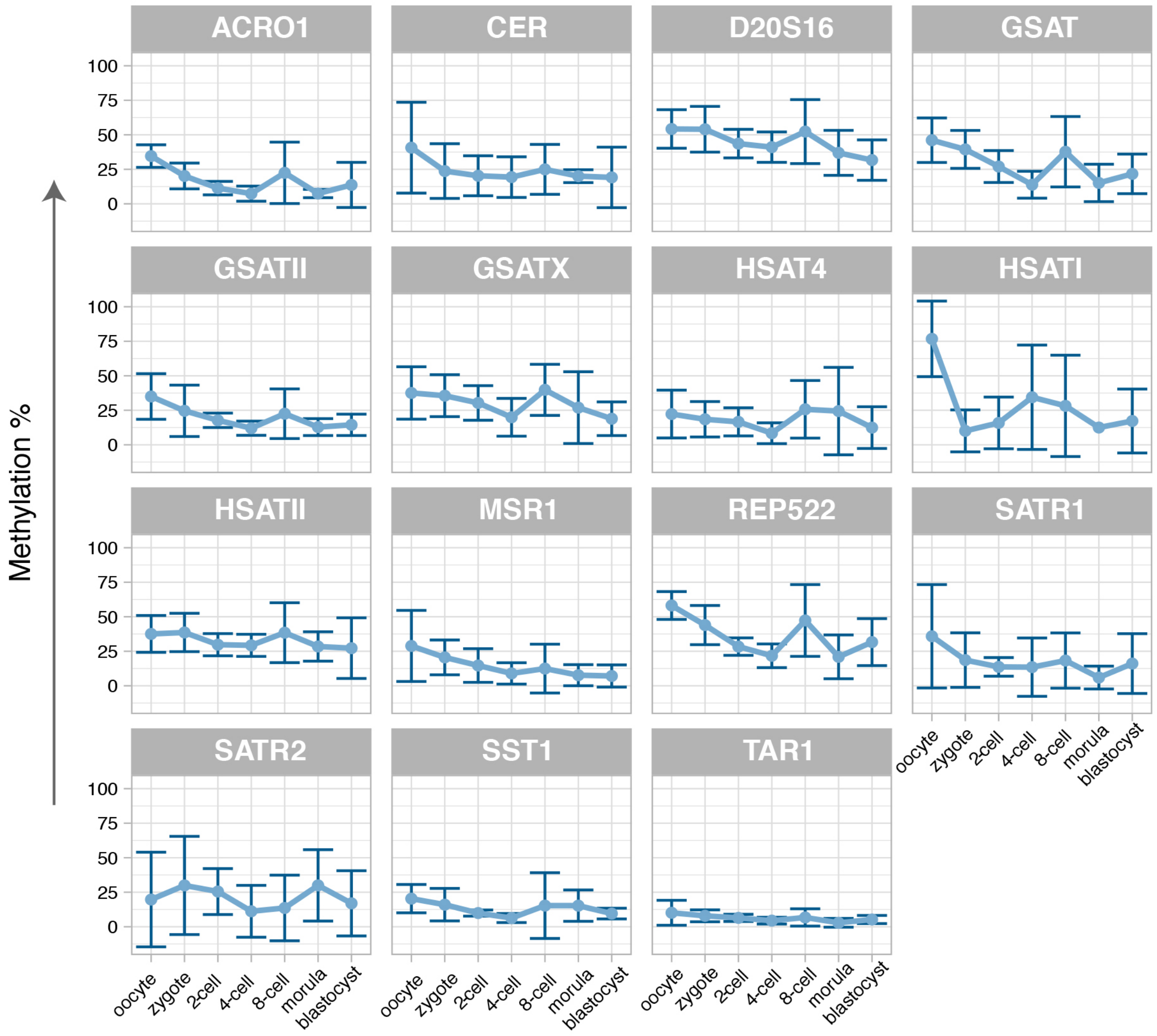

Figure S9. Line plots representing mean DNA methylation levels of different members of satellite repeat family. Error bars indicate the standard error of the mean.
